# Green Synthesis of Luminescent Gold-Zinc Oxide Nanocomposites: Cell Imaging and Visible Light–Induced Dye Degradation

**DOI:** 10.3389/fchem.2021.639090

**Published:** 2021-04-14

**Authors:** Kanika Bharti, Shahbaz Ahmad Lone, Ankita Singh, Sandip Nathani, Partha Roy, Kalyan K. Sadhu

**Affiliations:** ^1^ Department of Chemistry, Indian Institution of Technology Roorkee, Roorkee, India; ^2^ Department of Biotechnology, Indian Institution of Technology Roorkee, Roorkee, India

**Keywords:** nanocomposites, green synthesis, luminescence, photodegradation, redox chemistry

## Abstract

Green synthesis of gold-zinc oxide (Au-ZnO) nanocomposite was successfully attempted under organic solvent–free conditions at room temperature. Prolonged stirring of the reaction mixture introduced crystallinity in the ZnO phase of Au-ZnO nanocomposites. Luminescence properties were observed in these crystalline Au-ZnO nanocomposites due to *in situ* embedding of gold nanoparticles (AuNP) of 5–6 nm diameter on the surface. This efficient strategy involved the reduction of Au(III) by Zn(0) powder in aqueous medium, where sodium citrate (NaCt) was the stabilizing agent. Reaction time and variation of reagent concentrations were investigated to control the Au:Zn ratio within the nanocomposites. The reaction with the least amount of NaCt for a long duration resulted in Au-ZnO/Zn(OH)_2_ nanocomposite. X-ray photoelectron spectroscopy (XPS) confirmed the formation of Zn(OH)_2_ and ZnO in the same nanocomposite. These nanocomposites were reconnoitered as bioimaging materials in human cells and applied for visible light–induced photodegradation of rhodamine-B dye.

## Introduction

The development of luminescent AuNP has been an active area of material research over the past decade due to its potential applications from bench to clinical settings (Liu et al., 2013; Yu et al., 2019). Luminescent AuNP with 3 nm diameter showed a pH-dependent membrane adsorption property (Yu et al., 2011). Gold nanoclusters with small diameters and aggregated AuNP have also found to be luminescent in nature (Gan et al., 2016; Goswami et al., 2016; Saini et al., 2017; J. Wang et al., 2018a; Y. Wang et al., 2018b; Wu et al., 2019). In addition to imaging applications, deposition of gold nanoclusters or nanoparticles was successfully attempted on Ag@SiO_2_ or covalent organic framework for important functional activities such as circulating miRNA in human serum (Zhang Q. et al., 2019) or surface-enhanced Raman scattering (He et al., 2017), respectively. Building upon this past research, the present study developed a method to deposit luminescent AuNP with 5–6 nm diameter on the surface of Au-ZnO nanocomposites.

Au-metal oxide nanocomposites are known not only for their high surface-area-to-volume ratio but also for enhanced stability during their catalytic activity (Li et al., 2011; Ray and Pal, 2017; Wei et al., 2017; Cyganowski et al., 2019; Kauffman et al., 2019; Mageed et al., 2019). These hybrid nanomaterials possess unique optical, electronic, and magnetic properties, which are governed by their structural features, size, and compositional heterogeneities (Zu et al., 2015; Chamorro et al., 2016; Dutta Chowdhury et al., 2017; Lee et al., 2017; Song et al., 2017; Hu et al., 2018; Liu F. et al., 2019). A handful of synthetic methods of Au-metal oxide nanocomposites such as chemical vapor deposition, physical vapor deposition, hydrothermal method, spray pyrolysis, electrophoretic deposition, microwave-assisted thermal decomposition, magnetron sputtering, and spin coating are described in the literature (He et al., 2010; Shingange et al., 2016; Klug et al., 2017; Y. Wang et al., 2017a).

Considering the growing demand for Au-metal oxide–based nanocomposites for industrial and biomedical applications (Chen D. et al., 2015; Chen et al., 2017; Liu et al., 2018; Zhou et al., 2018; Gao et al., 2019; Jia et al., 2019; Zhang M. et al., 2019), there is a critical need to design a more facile and well-regulated synthetic route. Synthesis of metal-based nanocomposites by organic solvent–free conditions has drawn a lot of attention in the current decade (Li et al., 2013; Yao et al., 2013; Zhang et al., 2016; Qi et al., 2019; Ritchie et al., 2019). Au-ZnO nanocomposites, which are well known for a variety of applications, have been synthesized at high temperature in the presence of organic solvent (Yao et al., 2011; Tahir et al., 2013; Hang et al., 2016; Chang et al., 2017; Lupan et al., 2019). There are two reports, where AuNP was decorated on the crystalline ZnO nanorod surface at high temperature (Unlu et al., 2015; Ning et al., 2019). However, there is a lacuna of AuNP on the surface of Au-ZnO nanocomposites due to the synthetic challenges involving the two gold nanostructures simultaneously on the same material. In our synthetic methodology, the occlusion of AuNP of 5–6 nm diameter was successful for the first time on the surface of Au-ZnO nanocomposites in aqueous medium and at room temperature by the reduction of Au(III) salt with Zn(0) powder in the presence of AuNP as seed and NaCt as the stabilizing agent.

Au-ZnO nanocomposites with different Au:Zn compositions were prepared by varying the amount of AuNP seed, NaCt, and Zn metal powder. The reaction mixture in solution or the isolated nanocomposite materials in solid state were characterized by electronic absorption, XPS, FE-SEM (field emission scanning electron microscopy), energy dispersive X-ray (EDX), TEM (transmission electron microscopy), PXRD (powder X-ray diffraction), and surface charge analysis. All these characterizations of nanocomposites confirmed the key roles of AuNP seed, NaCt, and Zn metal in controlling Au:Zn ratio.

**SCHEME 1:**
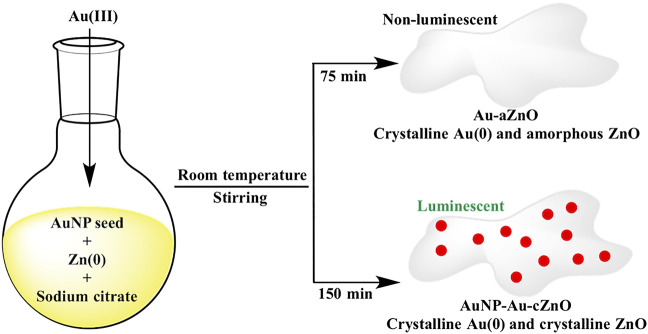
Synthetic route of Au-aZnO **(aZn1**-**aZn8)** and AuNP-Au-cZnO **(cZn1**-**cZn4)** nanocomposites in aqueous medium.

The redox reaction between Au(III) and Zn(0) was initially performed for 75 min, and this led to development of Au-ZnO nanocomposites (Au-aZnO, **aZn1**-**aZn8**) with ZnO in the amorphous phase (Scheme 1). However, when the same reaction was continued for 150 min, both Au and ZnO were found to be in crystalline phase without much variation in Au:Zn ratio. Interestingly, AuNP of 5–6 nm diameter was occluded on the surface of crystalline Au-ZnO nanocomposites (AuNP-Au-cZnO, **cZn1**-**cZn4**) during 150 min stirring of the reaction mixture (Scheme 1). Introduction of AuNP in AuNP-Au-cZnO showed a luminescent property with maximum emission at 496 nm after excitation of the samples at 436 nm. In comparison, Au-aZnO did not show any luminescence properties. This method opens up new avenues for fabricating Au-ZnO nanocomposites with different compositions under mild conditions. The excitation wavelength of the luminescent AuNP-Au-cZnO nanocomposites helped in the photodegradation of rhodamine-B in the presence of 455 nm light.

## Materials and Methods

**Materials.** The chemicals gold (III) chloride trihydrate and rhodamine-B were purchased from Sigma-Aldrich, and trisodium citrate dihydrate was purchased from Merck chemicals. Zinc powder was purchased from SISCO Research Laboratory. All glassware and stirrer bars were washed with freshly prepared aqua regia (mixture of 1:3 volume ratio of nitric acid:hydrochloric acid) and then with Millipore water and dried in an oven before use.

**Synthesis of gold nanoparticle seed.** AuNP seed (average size 19 ± 3 nm) solution with surface plasmon resonance (SPR) peak at 524 nm was prepared by the NaCt-based reduction method (Wuithschick et al., 2015). The concentration in terms of nanoparticle per ml was calculated as per methods given in the literature (Johonston, 2002; Lewis et al., 2006).

**Preparation of seed stock.** Seed solution was prepared by taking 200 µL as-synthesized gold nanoparticle and adding to it 19.8 ml of 1% (w/v) trisodium citrate dihydrate solution to make final volume to 20 ml.

Synthetic schemes and brief characteristics of all nanocomposites have been summarized in Supplementary Schemes S1, S2.

**Syntheses of the nanocomposites aZn1 to aZn5.** For zinc-gold nanocomposites, five different solutions were prepared. Each solution contained 20 mg (0.05 mmol) HAuCl_4_ in 200 ml of Millipore water. To these solutions, five different nanoparticles per mL (22.4 × 10^4^, 45 × 10^4^, 90 × 10^4^, 180 × 10^4^, and 900 × 10^4^) as seed were added, respectively, to produce **aZn1** to **aZn5**. In each of these five solutions, additional 2 ml 1% (w/v) solution of trisodium citrate dihydrate (0.07 mmol) and 20 mg zinc powder (0.3 mmol) were added. All these reaction mixtures were stirred for 75 min at room temperature followed by filtration.

**Syntheses of nanocomposites aZn6 and aZn7.** Two solutions contained 20 mg (0.05 mmol) HAuCl_4_ in 200 ml of Millipore water. In both the solutions, 2 ml 1% (w/v) solution of trisodium citrate dihydrate (0.07 mmol) and 200 mg (3 mmol) of zinc were added. Two different seeds, 900 × 10^4^/ml and 22.4 × 10^4^/ml (nanoparticle/ml), were added to produce **aZn6** and **aZn7**, respectively. The reaction mixtures were stirred at room temperature for 75 min and filtered.

**Syntheses of gold-zinc nanocomposites using variable citrate concentration.** Five different solutions were prepared. Each solution contained 20 mg (0.05 mmol) HAuCl_4_ in 200 ml of Millipore water. The addition of seed (nanoparticle/ml) was restricted to 22.4 × 10^4^/ml in each solution, and the reaction was stirred for 75 min. Five different amounts (0.2, 0.4, 0.6, 0.8, and 1.0 ml) of 1% (w/v) solution of trisodium citrate dihydrate were added followed by addition of 20 mg zinc powder (0.3 mmol). The reaction mixtures were stirred for 75 min and filtered. The filtered solutions were characterized by UV-visible spectroscopy.

**Synthesis of nanocomposite aZn8.** One solution contained 20 mg (0.05 mmol) HAuCl_4_ in 200 ml of Millipore water. To this were added seed (nanoparticle/ml) of 900 × 10^4^/ml and 0.021 mmol of trisodium citrate dihydrate followed by addition of zinc (3.0 mmol) powder to obtain **aZn8**. The reaction mixture was stirred at room temperature for 75 min and filtered.

**Synthesis of cZn1.** 20 mg (0.05 mmol) HAuCl_4_ was added to 200 ml of Millipore water. To the solution, seed of 900 × 10^4^/ml (nanoparticle/ml) was added during stirring followed by 2 ml 1% (w/v) solution of trisodium citrate dihydrate (0.07 mmol) and 20 mg zinc powder (0.3 mmol) to obtain **cZn1**. Reaction mixture was stirred for 150 min at room temperature followed by filtration.

**Synthesis of cZn2.** 20 mg (0.05 mmol) HAuCl_4_ was added to 200 ml of Millipore water. Then, during stirring, seed of 900 × 10^4^/ml (nanoparticle/ml) was added followed by 2 ml 1% (w/v) solution of trisodium citrate dihydrate (0.07 mmol) and 200 mg (3 mmol) of zinc metal powder to obtain **cZn2**. The reaction mixtures were stirred at room temperature for 150 min and filtered.

**Synthesis of cZn3.** 20 mg (0.05 mmol) HAuCl_4_ was added to 200 ml of Millipore water. Then, during stirring, seed of 22.4 × 10^4^/ml (nanoparticle/ml) was added followed by 2 ml 1% (w/v) solution of trisodium citrate dihydrate (0.07 mmol) and 200 mg (3 mmol) of zinc metal powder to obtain **cZn3**. The reaction mixtures were stirred at room temperature for 150 min and filtered.

**Synthesis of cZn4.** 20 mg (0.05 mmol) HAuCl_4_ was added to 200 ml of Millipore water. Then, during stirring, seed of 900 × 10^4^/ml (nanoparticle/ml) was added followed by 600 μL 1% (w/v) solution of trisodium citrate dihydrate (0.021 mmol) and 200 mg (3 mmol) of zinc metal powder to obtain **cZn4**. The reaction mixtures were stirred at room temperature for 150 min and filtered.

**Yield of cZn1 to cZn4.** The bulk scale reactions were carried out with 100 mg (0.25 mmol) HAuCl_4_ in one batch. The other reagents were taken in the same ratio mentioned in the syntheses. After the filtration process, the filtrate was centrifuged and finally dried under vacuum to isolate the solid **cZn1** to **cZn4**. The yields of **cZn1**, **cZn2**, **cZn3**, and **cZn4** were 45, 57, 54, and 43 mg, respectively. These solids were stored at room temperature in dark for further application and characterization.

**Synthesis of ZnO.** ZnO was prepared by the reported method of (Pourrahimi et al., 2014). 250 ml of 0.1 M zinc sulfate salt was stirred for 15 min at 60°C. After 15 min, 250 ml 0.25 M NaOH solution was heated separately at 60°C and added to the zinc sulfate solution during stirring. The reaction was continued with stirring for 60 min. The white precipitate obtained was kept for calcination at 400°C for 3 h in a muffle furnace.

## Methods

**Absorption spectroscopy.** Nanocomposite solutions were characterized using a UV-Vis spectrometer (UV-1601, Shimadzu). Absorbance measurement was taken over 400–800 nm wavelength range.

**Transmission electron microscopy (TEM).** The TEM images of nanocomposites and SAED patterns were obtained using FEI, Technai G2 20 S-TWIN. Image J software was used to analyze the average diameter of GNPs.

**Field emission scanning electron microscopy (FE-SEM).** The FE-SEM (Carl ZEISS Ultra plus Gemini, Germany) images were employed to analyze the morphological features. Energy-dispersive X-ray (EDX) and EDX-mapping of the nanocomposites were performed to find the composition.

**X-ray photoelectron spectroscopy (XPS).** XPS experiments were performed with PHI 5000 Versa Prob II, FEI Inc., and a C60 sputter gun has been used for characterization. The chemical states of the gold nanoparticles were characterized by XPS with monochromatized Al K(α) excitation (h = 1486.6 eV). The C 1s (284.8 eV) was used as a reference to calibrate the peak X-ray photoelectron spectroscopy (XPS) with Auger electron spectroscopy (AES) module positions of the elements.

**Powder X-ray diffraction (PXRD).** PXRD was performed using Bruker-D8 advance with an X-ray source, a 2.2 kW Cu anode, and an accelerating voltage of 40 kV.

**Luminescence measurement.** Luminescence of nanocomposites **cZn1**, **cZn2**, **cZn3**, and **cZn4** was measured using a Synergy microplate reader (Biotek United States) instrument, within the range of 470–700 nm with λ_ex_ = 436 nm. The relative quantum yield was calculated with respect to fluorescein in 0.1 M NaOH (Q.Y. = 0.95) as reference (Brouwer, 2011) for comparison. The excitation spectra were measured using the same instrument within the range of 380–460 nm with λ_em_ = 496 nm.

**Surface charge measurement.** Surface charge measurements were obtained using a Zetasizer Nano ZS90 (Malvern Instruments). DTS applications 7.03 software was used to analyze the data.

**Time-resolved fluorescence measurement.** Fluorescence decay of fluorescein was estimated using the TCSPC system from Horiba Jobin Yvon FluoroHub Instrument, with λ_ex_ = 435 nm and λ_em_ = 496 nm. Data analysis was performed with DAS6 software. The decay time data were analyzed using exponential sum, employing a nonlinear least squares reconvolution analysis. Average fluorescence lifetimes were calculated as Σα_i_τ_i_^2^/Σα_i_τ_i_ with normalized α_I_ (Lakowicz, 2006).

**Photocatalytic degradation of rhodamine-B using 455 nm LED or 254 nm UV light.** Nanocomposites **cZn1**, **cZn2**, **cZn3**, and **cZn4** (10 mg) were dispersed in 18 ml deionized water, separately sonicating them for 10 min. To these, 2.0 ml of 1.0 × 10^−4^ M rhodamine-B stock solution was added and stirred in dark for 30 min before irradiation in 455 nm LED light or 254 nm UV light. The samples were then kept under light; absorption spectra were recorded at regular intervals of time. The photocatalysis experiments were carried out at pH 7 and at room temperature. A decrease in absorption maximum at 556 nm was observed with photocatalytic degradation of the rhodamine-B dye.

**Cell viability assay.** To determine the cytotoxic effect of the test materials, MTT (3-(4,5-dimethylthiazol-2-yl)-2,S- diphenyltetrazolium bromide) assay was carried out according to the protocol reported elsewhere. In brief, the cells (5000 cells/200 µL/well) were seeded in a 96-well plate. Nanocomposite suspension at 50 μg/ml concentrations was added to the monolayer in triplicate and incubated for 2, 4, 8, 12, and 24 h. Then, after the addition of 20 μL of 5 mg/ml MTT (Sigma-Aldrich, MO, United States) reagent, the cells were allowed to incubate for another 4 h at 37°C. The formazan crystals formed inside the cells were then solubilized by adding 200 μL of DMSO (HiMedia, Mumbai, India) to each well. The viable cells that showed the formation of violet crystals were quantified at 570 nm using a microplate reader (Omega fluostar, BMG Labtech Ltd., Germany). The cell cytotoxicity was expressed as percentage cell viability in comparison to the control group.

**Fluorescence microscopy.** The internalization of nanocomposites was monitored at 2 h, and the images were captured using a fluorescence microscope (Evos Floid cell imaging station, Invitrogen, United States) under ×200 magnification. In brief, HEK293 cells (5000 cells/200 µL/well) were seeded in a 96-well plate and allowed to adhere for 24 h. Then, nanocomposites (50 μg/ml) were incubated with the cells for 2 h, and the images were captured after washing with PBS.

## Results and Discussion

**Synthesis of optimized materials of amorphous and crystalline Au-ZnO nanocomposites (**Au-aZnO, **aZn5-aZn8,** and AuNP-Au-cZnO, **cZn1**-**cZn4).** Two different types of nanocomposites on the basis of Zn powder as reducing agent were synthesized from aqueous solution of HAuCl_4_ by variation of AuNP seed, Zn powder, and NaCt as stabilizing agents as mentioned in Table 1. In order to develop the crystalline nature in the ZnO part of the newly synthesized nanocomposites (**cZn1**-**cZn4**) by maintaining green synthetic methodology (Ning et al., 2019), we focused on the reaction time duration instead of introducing organic solvent or high temperature. The crystallinity in the nanocomposites was obtained by increasing the reaction time twice to that of the initial reactions. During all the syntheses, a reducing agent was added at last. The pH of the solution before the addition of Zn powder varied within the range of 6.8–7.0, while the pH of the solution increased within the range of 7.3–7.8 after stirring for either 75 min or 150 min. This pH range helped in the stabilization of the basic Au-ZnO nanocomposite in the aqueous medium. The procedure was adapted on the basis of redox reaction between Au(III) and Zn(0), which does not require a basic or acidic medium. As ZnO is amphoteric in nature, there is a chance that zinc salt will form at a pH that is too low and that zinc hydroxide will form at a high pH (Degen and Kosec, 2000).

**TABLE 1:** Summary of Au-ZnO nanocomposites based on EDX analysis from FE-SEM.

Au(III) (mmol)	Sodium citrate (mmol)	Zn (mmol)	Seed concentration (×10^4^ per ml)	Zn:Au in nanocomposite	
				Reaction time 75 min	Reaction time 150 min
0.05	0.07	0.3	900	0.1:1 (**aZn5**)	0.1:1 (**cZn1**)
0.05	0.07	3	900	1.0:1 (**aZn6**)	1.1:1 (**cZn2**)
0.05	0.07	3	22.5	0.9:1 (**aZn7**)	1.1:1 (**cZn3**)
0.05	0.021	3	900	0.5:1 (**aZn8**)	0.5:1 (**cZn4**)

The concentration of seed was varied within the range of 22.4 × 10^4^ per ml to 900 × 10^4^ per ml. There is a blueshift in the absorbance (marked with an arrow in Supplementary Figure S1) from **aZn1** to **aZn5**. However, all these redshifted absorbance peaks with respect to seed are either due to the growth of seed **aZn1** or due to the incorporation of ZnO layer in the Au-ZnO composite **aZn5** (Viter et al., 2015). We performed a series of experiments (Supplementary Figure S1) to generate the SPR peaks for gold nanomaterials by varying the zinc amount. We have noticed that a minimum of 6 equivalents of zinc is required to produce stable SPR peak and the peak intensity gets saturated in presence of 60 equivalents. The presence of 60 equivalents of reducing agent with respect to HAuCl_4_ did not show much difference in Zn amount among the synthesized nanocomposites (**aZn6**, **aZn7**, **cZn2**, and **cZn3**). Lowering down the concentration of reducing agent from 60 equivalents to 6 equivalents drastically decreased the incorporated Zn amount (*vide infra*) within the nanocomposites **aZn5** or **cZn1**. The treatment of 30% stabilizing agent with respect to the synthesis of **aZn6** or **cZn2** showed a 50% decrease in the incorporated Zn amount in the nanocomposites **aZn8** or **cZn4**.

**Synthesis of remaining Au-ZnO nanocomposites (**Au-aZnO, **aZn1**-**aZn4) and characterization of aZn1**-**aZn8.** Room temperature reaction in aqueous medium with variable AuNP seed concentration in the presence of NaCt (0.07 mmol) as stabilizing agent and Zn (0.3 mmol) as reducing agent for HAuCl_4_ (0.05 mmol) produced a violet color in different solutions (Supplementary Figure S1) within 75 min. The redshift observed in the SPR band of **aZn1** (λ_max_ = 575 nm) formed using 22.5 × 10^4^ particles/ml in comparison to seed is due to formation of ZnO. This type of shift due to the formation of the ZnO layer on Au-film was reported in the literature (Viter et al., 2015). Reaction with 900 × 10^4^/ml seed concentration showed **aZn5** with enhanced coloration with blueshift (λ_max_ = 555 nm) compared to **aZn1**. However, this absorption exhibited clear redshift with respect to seed particle (Yu et al., 2005). In absence of AuNP seed, there was no SPR absorbance in the reaction mixture even after 3 h of reactions between HAuCl_4_ and Zn powder in the presence of NaCt.

The formations of Au-ZnO nanocomposites were confirmed with TEM, FE-SEM images, and EDX analysis of **aZn1** to **aZn5** (Supplementary Figures S2, S3). In the synthesis process, the metal powder was engaged in redox reaction between Au(III)/Au(0) and Zn(II)/Zn(0) couple, in addition to playing the role of simple metal source, thereby leading to the formation of Au-ZnO nanocomposites. Although the incorporation of Zn in the respective nanocomposite **aZn1** was sparse, we were successful in increasing the Au:Zn ratio up to 1:0.1 (weight percentage) in **aZn5**.

An increase in the concentration of Zn (3 mmol) led to rapid (∼1 min) appearance of violet color in the reaction mixture for **aZn6** (Supplementary Figure S4). In PXRD (Supplementary Figure S5), only the characteristic peaks of Au(0) were observed for **aZn6**, suggesting the amorphous (X. Wang et al., 2017b) nature of ZnO in Au-aZnO nanocomposites. An enhancement of approximately 10 times the Zn content in **aZn6** (Au:Zn = 1:1) was confirmed by FE-SEM and EDX analysis (Supplementary Figure S6). No visible color generation in the absence of Zn powder (Supplementary Figure S4) affirmed the role of the metal powder in the redox process. The variation of seed concentration involving higher amount of Zn powder did not show any appreciable shift in the plasmonic bands (Supplementary Figure S4). Nanocomposite **aZn7** synthesized using 3 mmol Zn powder with 22.4 × 10^4^/ml seed concentration resulted in Au:Zn = 1:0.9 (Supplementary Figure S7).

In order to rule out the competitive reduction of Au(III) by NaCt, the amount of the same was decreased from 0.035 to 0.007 mmol for Au-aZnO nanocomposites preparation at a constant seed concentration of 22.4 × 10^4^/ml and Zn powder of 0.3 mmol (Supplementary Figure S8). We performed the synthesis of nanocomposites with different concentrations of sodium citrate. We observed that a stable SPR peak was generated in the presence of 0.021 mmol of sodium citrate. This result suggested that a threshold amount of NaCt (0.021 mmol) was essential as the stabilizing agent in the reduction process to synthesize the Au-aZnO nanocomposites. Lowering the NaCt concentration from 0.07 to 0.021 mmol with 900 × 10^4^/ml led to the lowering of Zn incorporation in Au-ZnO nanocomposites, Au:Zn = 1:0.5 in **aZn8** (Supplementary Figures S9, S10). The chemical compositions of the Au-aZnO nanocomposites obtained from EDX analysis were further confirmed by XPS analysis. This also reveals the oxidation states of different elemental species present in Au-aZnO nanocomposites (Supplementary Figure S11). Binding energy studies confirmed the peaks of Au 4f_7/2_ (Fuggle et al., 1977), Zn 2p_3/2_ (Strohmeier and Hercules, 1984), and O 1s (Tan et al., 1990) at 83.0, 1021.4, and 530 eV, respectively, in these nanocomposites. These Au-aZnO nanocomposites were found to be nonluminescent in nature unlike the previously reported emissive ZnO nanoparticles (Tang et al., 2010; Jangir et al., 2017; Raji and Gopchandran, 2017).

**FIGURE 1:**
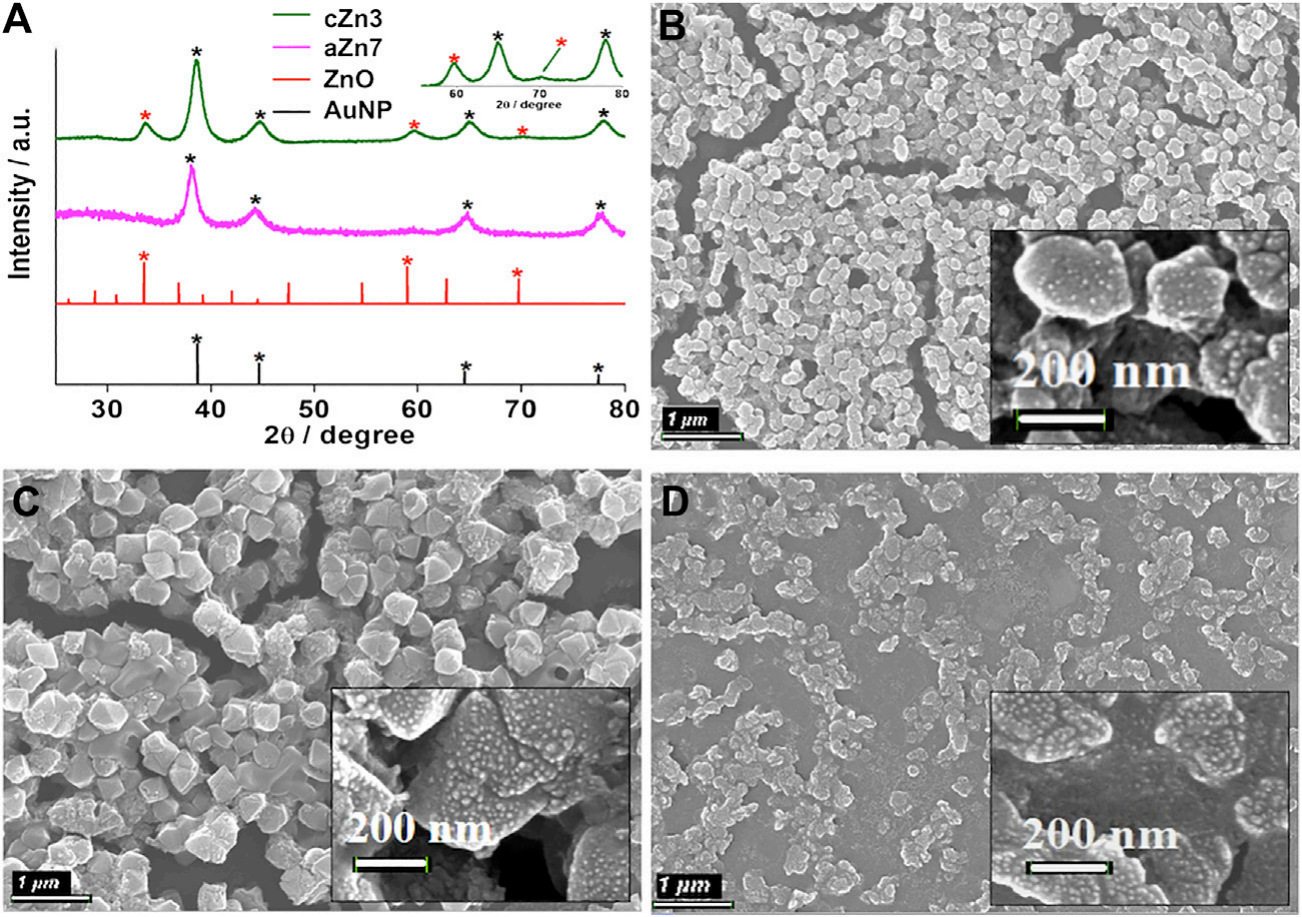
**(A)** XRD of **aZn7** and **cZn3** compared with the AuNP (JCPDS no. 00–004–0784) and ZnO (JCPDS no. 00–021–1486); FE-SEM images of AuNP-Au-cZnO nanocomposites: **(B) cZn2**, **(C) cZn3**, and **(D) cZn4**. The scale bar for B–D: 1 µM and the scale bar for inset images: 200 nm.

**Characterization of crystalline Au-ZnO nanocomposites (**AuNP-Au-cZnO, **cZn1**-**cZn4).** EDX analyses of the samples after the reaction with a longer duration resulted in a similar amount of ZnO incorporation in the nanocomposites (Supplementary Figures S12–S15). However, crystallinity was introduced (Figure 1A; Supplementary Figure S16) in both the Au and ZnO components of the **cZn2**, **cZn3**, and **cZn4** nanocomposites. In the case of **cZn1**, the amount of ZnO was very less, which restricted visualization of the crystallinity of ZnO in this sample by PXRD. The distinct difference in the PXRD data for the samples **cZn2** to **cZn4** confirmed the additional peaks due to crystalline ZnO in these nanocomposites. XRD peak positions (in degrees) and their corresponding FWHM values (in degrees) are shown in Supplementary Table S1. In XRD patterns (Figure 1; Supplementary Figure S16), there is a small redshift as compared to ZnO (JCPDS no. 00-021-1486 and Caglar et al., 2009) due to the inclusion of gold in nanocomposites. This interaction of gold and zinc oxide in our nanocomposites has been further supported with more FWHM values compared to bare ZnO.

In the case of absorption studies, the nanocomposites **cZn1** to **cZn4** showed redshift in SPR peak (λ_max_ at ∼565 nm, Supplementary Figure S17) with respect to AuNP seed solution. The absorption peak at 565 nm was observed in the case of **cZn1**. However, in the case of **cZn4**, a broad flat peak was observed around a wide range of 500–700 nm. The changes in absorbance spectra with respect to **cZn2** are due to variation of seed nanoparticle/ml (**cZn3**), reducing agent (**cZn1**), and stabilizing agent (**cZn4**). These variations in the experimental conditions result in the different compositions of zinc to gold ratio in **cZn1** to **cZn4** (Table 1). There is strong interface damping of the surface plasmon due to the interaction with ZnO. The broadening and decrease of the peak intensity of the plasmonic band from **cZn1** to **cZn2** are due to an increase in ZnO layers as compared to gold seed. It indicates a strong charge carrier interaction at the interface between Au and ZnO (Gogurla et al., 2020). The slight variation in the absorbance in **cZn2** and **cZn3** is due to difference in the seed nanoparticle/ml for these syntheses. The broad and low intense SPR peak in **cZn4** is due to the less amount of stabilizing agent, which is essential to stabilize the AuNP and its SPR absorbance. The FE-SEM image of **cZn1** exhibited octahedral shape nanostructure (Supplementary Figure S18). The other FE-SEM images (Figures 1B–D) demonstrated that structure and shape morphologies were tuned in all four cases **cZn1** to **cZn4** with respect to **aZn5**, **aZn6**, **aZn7**, and **aZn8**. Interesting surface morphologies with clear appearances of dots were observed in FE-SEM images for the nanocomposites **cZn2** to **cZn4**. These dots were further clarified in the focused surfaces (Figures 1B–D insets) of the nanocomposites. The overall particle size of the AuNP-cZnO composite was increased up to 200 nm (Figure 1B) and to 400 nm (Figure 1D) due to the growth reaction between HAuCl_4_ and Zn powder at the edge of the AuNP seed as support and NaCt as a stabilizing agent.

**FIGURE 2:**
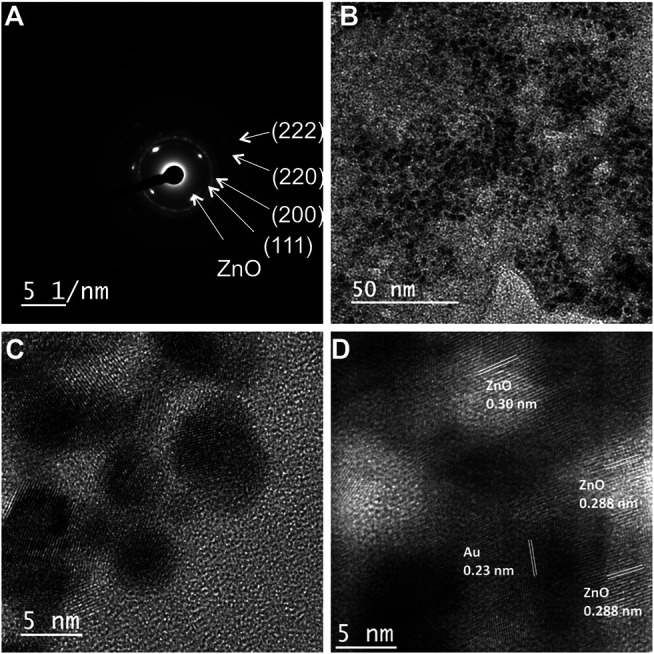
**(A)** SAED image of **cZn4**; **(B)** TEM image and **(C)** HR-TEM showing 5–6 nm AuNPs on nanocomposite **cZn4**, **(D)** HR-TEM image showing fringe lines corresponding to both Au and ZnO in **cZn4**.

In order to clarify the crystalline nature of both Au and ZnO in the nanocomposites, selected area electron diffraction (SAED) pattern was recorded. In the case of **cZn4**, the SAED patterns confirmed the presence of crystalline Au and ZnO in the same sample (Figure 2A). The diffraction patterns for Au planes were similar in **cZn1** to **cZn4**. The difference in the diffraction pattern is due to ZnO. The calculated distances between ZnO planes from diffraction SAED pattern are 0.29, 0.34, 0.30, and 0.28 nm for **cZn1**, **cZn2**, **cZn3**, and **cZn4**, respectively. This change in the ZnO plane from **cZn2** to **cZn4** is due to the additional incorporation of gold nanoparticles in these nanocomposites. Furthermore, the TEM image of **cZn4** (Figure 2B) corroborated the formation of small size AuNPs on the surface of AuNP-Au-cZnO nanocomposites. The aforementioned result confirmed the generation of AuNP on the nanocomposite surface via the reduction of Au(III) by Zn(0) powder. The high-resolution TEM (HR-TEM) image of the surface confirmed the occlusion of AuNP of 5–6 nm diameter (Figure 2C) on the **cZn4** nanocomposite surface. This type of Au-ZnO nanocomposite surfaces (Supplementary Figure S19) was also observed for **cZn1**-**cZn3**. From Williamson Hall plot (Supplementary Figure S20) analysis, lattice strains are calculated, and values are 0.00253, 0.00224, 0.00418, and 0.00808 for **cZn1**, **cZn2**, **cZn3**, and **cZn4**, respectively. This trend in lattice strain is due to the change in crystal packing (confirmed from HR-TEM and SAED patterns) associated with the degree of disorders (Gogurla et al., 2018).

During the synthesis of nanocomposites, 19 nm AuNP seeds did not convert to 5–6 nm AuNP, which was occluded *in situ* on the Au-ZnO surface. These 5–6 nm Au nanoparticles were obtained directly during the reduction of HAuCl_4_ by Zn powder in the synthetic process. This generation of 5–6 nm AuNP was not even due to Ostwald ripening as this type of ripening process was involved in the postsynthetic step (Jang et al., 2012). Moreover, Ostwald ripening showed very less enhancement with higher particle size such as 15 nm AuNP (Pattadar and Zamborini, 2019).

Another HR-TEM image of the **cZn4** surface, on which AuNP occlusion was observed, confirmed lattice fringes corresponding to both Au and ZnO. The d-spacing of 0.23 and 0.20 nm (Figure 2D; Supplementary Figure S21) revealed the presence of Au (111) and Au (200) planes (Guo et al., 2017). The interplanar spacings of 0.288 and 0.30 nm were found consistent with ZnO, which is in agreement with the XRD spectral peaks according to Joint Committee on Powder Diffraction and Standards (JCPDS no. 00-021-1486; Pradhan and Leung, 2008). Lattice mismatches between Au and ZnO were also observed in this HR-TEM image.

**FIGURE 3:**
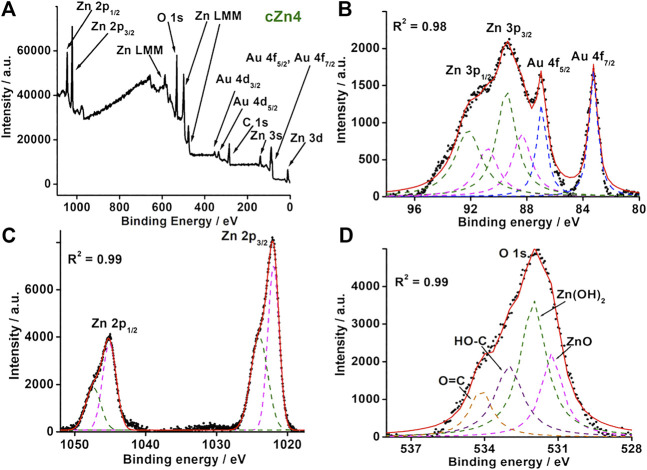
**(A)** Survey XPS spectrum and **(B–D)** high-resolution XPS spectra for Au 4f, Zn 3p, Zn 2p, and O 1s of nanocomposite **cZn4**.

In order to find the detailed composition of **cZn1**-**cZn4**, the XPS survey scans for all the samples were performed (Figure 3A; Supplementary Figures S22–S24). These survey scans indicated the presence of Au, C, O, and Zn in the nanocomposites. For nanocomposite **cZn1** (Supplementary Figure S22), carbon 1s spectra showed two peaks at 284.8 and 288.7 eV due to aliphatic carbon of –COO group (Di Mauro et al., 2017). The high-resolution scan XPS spectra of Zn illustrated Zn 2p_3/2_ and Zn 2p_1/2_ peak at 1021 and 1044.8 eV, which were reported for the ZnO system. The O 1s spectrum shows a peak at 531.68 eV due to O^2-^ species present in oxygen deficient regions of ZnO and 531.68 eV for C=O bond. Deconvulating high-resolution XPS spectra of Au showed a peak at 83.2, 86.9, 88.8, and 91.67 eV, which corresponded to Au 4f_7/2_, Au 4f_5/2_, Zn 3p_3/2_, and Zn 3p_1/2_, respectively. The binding energies for Au 4f_7/2_ and Au 4f_5/2_ were shifted slightly in comparison to Au(0) at 84 and 88 eV due to electron transfer from ZnO to Au (Gogurla et al., 2014). The XPS analyses for **cZn2** and **cZn3** (Supplementary Figures S23, S24) showed similar characteristics.

For nanocomposite **cZn4** (Supplementary Figure S25), carbon 1s spectra showed a similar trend to that of **cZn1**. The high-resolution XPS spectrum of Au (Figure 3B) was resolved into six peaks and out of these two corresponded to Au 4f_7/2_ (83.19 eV) and Au 4f_5/2_ (86.98 eV). The other four peaks at 88.35, 89.40, 90.80, and 92.18 eV were due to Zn 3p_3/2_ and 3p_1/2_ from ZnO and Zn(OH)_2_. High-resolution XPS spectrum for Zn (Figure 3C) was resolved into four peaks at Zn 2p_3/2_ (1022.9 eV) and Zn 2p_1/2_ (1045.12 eV) which correspond to ZnO and XPS peak at Zn 2p_3/2_ (1023.9 eV) and Zn 2p_1/2_ (1047.44 eV) contributing to Zn(OH)_2_ (Klug et al., 2017). The O 1s spectrum (Figure 3D) showed four peaks at 531.2, 532.0, 533.0, and 534.14 eV due to oxygen vacancies in ZnO and Zn(OH)_2_ and the oxygen atom of C-O, C=O bonds (Geng et al., 2018). The band gap due to zinc hydroxide compares to the band gap of ZnO (Karakawa et al., 2018). The ratio of two XPS peak areas of bare ZnO (Supplementary Figure S26) with nanocomposites suggests the maximum interaction between Au and ZnO in **cZn2** and **cZn4** (Supplementary Table S2).

FE-SEM, HR-TEM, and XPS together confirmed the occlusion of AuNP on the surface of Au-ZnO nanocomposites in the cases of **cZn2** and **cZn3**. In the case of **cZn4**, synthesized with the threshold amount of NaCt, AuNP occlusion took place on the surface of Au-ZnO/Zn(OH)_2_. The surface charges of **cZn1** and **cZn2** were found to be –3.78 and –0.33 mV (SupplementaryTable S3). These differences were due to the variation of electron transfer from ZnO to Au (Gogurla et al., 2014). In the case of **cZn3**, highly negative charge surface (-24.4 mV) was obtained due to the presence of less amount of seeds (nanoparticle/ml) in comparison to **cZn2**. For **cZn4**, the electron transfer from ZnO to Au was less due to the presence of Zn(OH)_2_ in the nanocomposite. This reflected in the slightly positive surface of **cZn4** (0.36 mV) nanocomposites.

**FIGURE 4:**
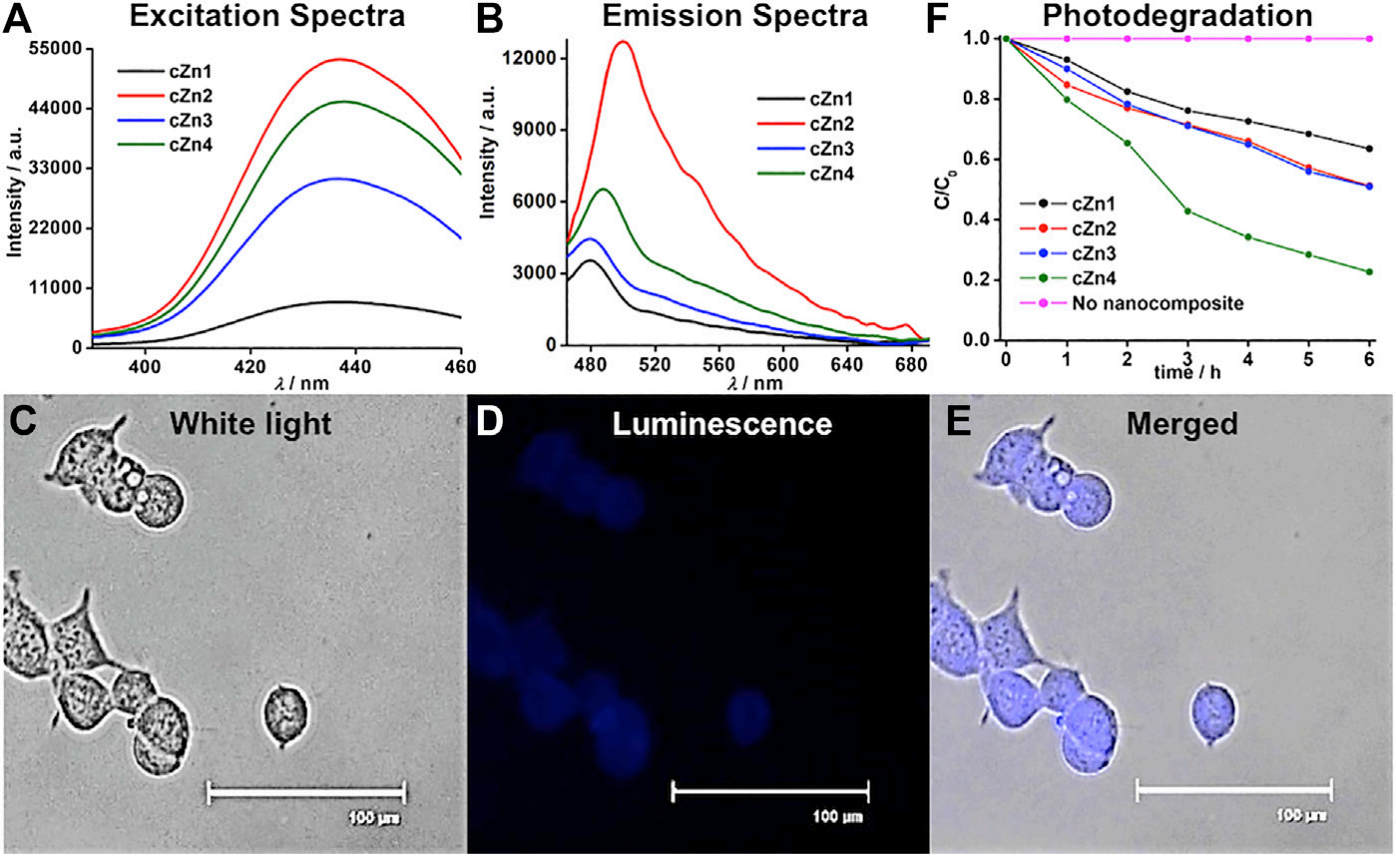
**(A)** Excitation, **(B)** emission spectra of nanocomposites **cZn1**-**cZn4**, **(C–E)** imaging of **cZn2** in HEK293 cells, where cells were excited at 390/40 nm and emissions were monitored with 446/33 blue filter for luminescence image, scale bar: 100 μm, **(F)** photodegradation of rhodamine-B in presence of nanocomposites **cZn1**-**cZn4**.

**Luminescent properties of crystalline Au-ZnO nanocomposites (cZn1**-**cZn4) and their applications.** The absorbance spectra of these nanocomposites showed peaks at 358 nm (Supplementary Figure S17). The excitation of sample **cZn2** at 350 nm produced dual emission peaks at 398 and 448 nm (Supplementary Figure S27A) with a broad tail up to 700 nm. The excitation spectra for 398 and 448 nm showed a peak at 358 nm (Supplementary Figure S27B), which was similar to the absorption peak. In order to find out the origin of the broadening, the deconvolution of the emission spectra was performed. This resulted in the emission of three additional peaks at 496, 547, and 590 nm. The emission around 398 nm was due to band-to-band transition of ZnO nanomaterial (Kuiri and Pramanik 2018). The excitation spectrum (Supplementary Figure S27B) for the emission at 448 nm showed a peak at 358 nm, which was due to the presence of ZnO in the nanocomposite (Liu et al., 2019). The last two emissions at 547 and 590 nm were due to oxygen vacancies in the ZnO nanocomposites. Reported ZnO nanostructures exhibited visible emissions within 420–569 nm by exciting at 370 nm (Khokhra et al., 2017) due to the zinc defects such as interstitials, natural and singly and doubly ionized interstitials. The emission peaks at 448, 547, and 590 nm by exciting the sample at 358 nm in our case are due to the point defect of ZnO. The remaining emission at 496 nm by excitation at 436 nm is due to the formation of 5–6 nm AuNP. The time-resolved fluorescence study (Supplementary Figure S28) in our case showed the similar trend of three decay components, which were similar to the previously reported emission from gold nanoclusters (Chattoraj and Bhattacharyya 2014). The quantum yields at 398, 448, 496, 547, and 590 nm wavelengths for **cZn2** were found to be 4.23, 3.48, 2.00, 0.81, and 0.56%, respectively. The excitation spectra for the other three emission peaks showed a peak at 436 nm (Figure 4A). The origin of this excitation peak at 436 nm was probably due to the energy transfer from the initial dual emission for *in situ* stabilized AuNP on Au-ZnO nanocomposites. The maximum emission from **cZn2** was observed at 496 nm by exciting the aqueous suspension at 436 nm (Figure 4B). In order to avoid excitation in the UV region at 358 nm, we chose 436 nm as excitation wavelength for our further studies. We checked the luminescence property of **cZn1** to **cZn4** by measuring the emission and excitation spectra (Figures 4A,B) in aqueous suspension. The quantum yields of these broad emissions for **cZn1**, **cZn3**, and **cZn4** were found to be 0.27, 0.67, and 0.95%, respectively. The poor quantum yield was due to the mixing of emission from ZnO nanocomponent with the excitation peak due to 5–6 nm AuNP on the AuNP-cZnO surface. The difference in emission behavior is mostly due to the formation of small size AuNP and this type of emission is highly dependent on the size and shape of gold nanoparticles (Chen et al., 2015). The emission maxima vary with Zn powder, AuNP seed, and NaCt concentration as reflected by **cZn1**, **cZn3**, and **cZn4**, respectively for **cZn2**.

We tested the fluorescence imaging of **cZn1**-**cZn4** nanocomposites in HEK293 cells. The cells were treated with **cZn1**-**cZn4** (50 μg/ml) for 2 h (at a cell viability of almost 85%) and fixed on a glass slide with paraformaldehyde reagent. The HEK293 cell nuclei and cytoplasm showed bright emission (Figures 4C–E; Supplementary Figures S29, S30, blue color for visualization) for **cZn2**-**cZn4**. **cZn1** did not show any prominent luminescent image due to poor quantum yield. The MTT assays were performed with **cZn1**-**cZn4** on the test cell line after incubation for up to 24 h and found to be nontoxic up to 2 h at a concentration of 50 μg/ml (*p* < 0.5, Supplementary Figure S31).

These excitation spectra in the visible region for the nanocomposites triggered the photodegradation of rhodamine-B in presence of visible light (Figure 4F; Supplementary Figure S32) unlike the recent report with 254 nm light for Au-ZnO nanocomposites. We carried out the photodegradation of rhodamine-B in presence of 455 nm LED. This wavelength was close to the excitation spectrum (Supplementary Figure S27B) from 5–6 nm AuNP in the AuNP-cZnO nanocomposites. This emission at 496 nm was stable even after 455 nm LED light illumination for 1 h. **cZn4** showed the best degradation performance with the rate constant 0.25 h^−1^ (Table 2). The nonradiative decay rate constants (Supplementary Figure S28) were in a similar range for all the four samples. However, the radiative decay rate constant for **cZn4** was maximum and at least two times compared to the nearest radiative decay rate constant for **cZn3**. The photodegradation studies with visible 455 nm light were dependent on the emission properties originating from 5–6 nm AuNP. The control experiments with **aZn5**-**aZn8** nanocomposites without 5–6 nm AuNP showed no photodegradation of the dye in the presence of 455 nm light. The dye degradation rate constants (Table 2) in the presence of 254 nm light were much less in comparison to 455 nm LED probably due to the inherent excitation maxima at 436 nm in these nanocomposites. ZnO-based rate constants (Supplementary Figure S32; Table 2) for rhodamine dye degradation are 0.02 h^−1^ and 0.04 h^−1^ by irradiation at 455 and 254 nm light, respectively. These rate constants were less in comparison to the rate constants with Au-ZnO nanocomposites.

**TABLE 2:** Rate constants for photodegradation of rhodamine-B.

Nanocomposite	Rate constant (h^−1^)	
	λ = 455 nm	λ = 254 nm
**cZn1**	0.08	0.04
**cZn2**	0.11	0.07
**cZn3**	0.11	0.05
**cZn4**	0.25	0.11
**ZnO**	0.02	0.04

## Conclusion

In conclusion, we demonstrated facile room temperature synthesis of crystalline Au-ZnO nanocomposites by metal mediated redox reaction using water as a solvent. The synthetic modification successfully helped in the development of luminescent properties via the occlusion of AuNP on the crystalline Au-ZnO/Zn(OH)_2_ nanocomposite surface. These luminescent nanocomposites were successfully applied in the visible light–induced photodegradation of rhodamine-B dye. Currently, we are working on the occlusion of other luminescent materials on the nanocomposite surface.

## Data Availability

The original contributions presented in the study are included in the article/Supplementary Material; further inquiries can be directed to the corresponding author.
