# Supplementary material for: Green Synthesis of Luminescent Gold-Zinc Oxide Nanocomposites: Cell Imaging and Visible Light–Induced Dye Degradation
Source: Front Chem. 2021 Apr 14;9:639090. doi: 10.3389/fchem.2021.639090 (PMC8080447; doi:10.3389/fchem.2021.639090)
Supplement: Supplementary file 1 [file DataSheet1.pdf]

Green synthesis of luminescent gold-zinc oxide nanocomposites: cell imaging and visible light induced dye-degradation

Kanika Bharti,[1] Shahbaz Ahmad Lone,[1] Ankita Singh,[1] Sandip Nathani,[2] Partha Roy,[2] and Kalyan K. Sadhu[1,*]

[1]Department of Chemistry, Indian Institution of Technology Roorkee, Roorkee 247667, Uttarakhand, India
[2]Department of Biotechnology, Indian Institution of Technology Roorkee, Roorkee 247667, Uttarakhand, India

Correspondence:
Kalyan K. Sadhu
sadhu@cy.iitr.ac.in

Table of content

Scheme S1-S2……………………………………………………………………………… 2

Figure S1-S2……………………………………………………………………………..... 3

Figure S3-S4……………………………………………………………………………..... 4

Figure S5-S7………………………………………………………………………………. 5

Figure S8-S10…………………………………………………………………………...... 6

Figure S11-S14……………………………………………………………………………. 7

Figure S15-S17……………………………………………………………………………. 8

Figure S18-S19……………………………………………………………………………. 9

Figure S20-S21……………………………………………………………………………. 10

Figure S22………………………………………………………………………………… 11

Figure S23………………………………………………………………………………… 12

Figure S24-S25……………………………………………………………………………. 13

Figure S26-S28……………………………………………………………………………. 14

Figure S29-31……………………………………………………………………………... 15

Figure S32……………………………………………………………………………….... 16

Supporting Table S1-S2………………………………………………………………… 17

Supporting Table S3……………………………………………………………………... 18

aZn1

Experimental condition		Result	
Seed (nanoparticles/mL)	22.4×10^4	Zn:Au	0.07:1
Reducing agent (mmol)	0.3		
Stabilising agent	0.07		
Au(III)	0.05		
Time (min)	75		

Seed ↑ — Increasing seed amount →

aZn5

Experimental condition		Result	
Seed (nanoparticles/mL)	900×10^4	Zn:Au	0.1:1
Reducing agent (mmol)	0.3		
Stabilising agent	0.07		
Au(III)	0.05		
Time (min)	75		

Zinc ↑ — Increasing amount of reducing agent ↓

aZn6

Experimental condition		Result	
Seed (nanoparticles/mL)	900×10^4	Zn:Au	1.0:1
Reducing agent (mmol)	3.0		
Stabilising agent	0.07		
Au(III)	0.05		
Time (min)	75		

← Seed ↓ — Decreasing seed amount

aZn7

Experimental condition		Result	
Seed (nanoparticles/mL)	22.4×10^4	Zn:Au	0.9:1
Reducing agent (mmol)	3.0		
Stabilising agent	0.07		
Au(III)	0.05		
Time (min)	75		

Nact ↓ — Decreasing amount of stabilising agent →

aZn8

Experimental condition		Result	
Seed (nanoparticles/mL)	900×10^4	Zn:Au	0.5:1
Reducing agent (mmol)	3.0		
Stabilising agent	0.021		
Au(III)	0.05		
Time (min)	75		

Scheme S1: Flow chart for syntheses of Au-amorphous ZnO nanocomposites.

cZn1

Experimental condition		Result	
Seed (nanoparticles/mL)	900×10^4	Zn:Au	0.1:1
Reducing agent (mmol)	0.3	λ_{em} (max, nm)	480
Stabilising agent	0.07		
Au(III)	0.05	Quantum yield	0.27%
Time (min)	150		

Zinc ↑ ↓

cZn2

Experimental condition		Result	
Seed (nanoparticles/mL)	900×10^4	Zn:Au	1.1:1
Reducing agent (mmol)	3.0	λ_{em} (max, nm)	500
Stabilising agent	0.07		
Au(III)	0.05	Quantum yield	2.00%
Time (min)	150		

← Seed ↓

cZn3

Experimental condition		Result	
Seed (nanoparticles/mL)	22.4×10^4	Zn:Au	1.1:1
Reducing agent (mmol)	3.0	λ_{em} (max, nm)	480
Stabilising agent	0.07		
Au(III)	0.05	Quantum yield	0.67%
Time (min)	150		

Nact ↓ →

cZn4

Experimental condition		Result	
Seed (nanoparticles/mL)	900×10^4	Zn:Au	0.5:1
Reducing agent (mmol)	3.0	λ_{em} (max, nm)	488
Stabilising agent	0.021		
Au(III)	0.05	Quantum yield	0.95%
Time (min)	150		

Scheme S2: Flow chart for syntheses of Au-crystalline ZnO nanocomposites.

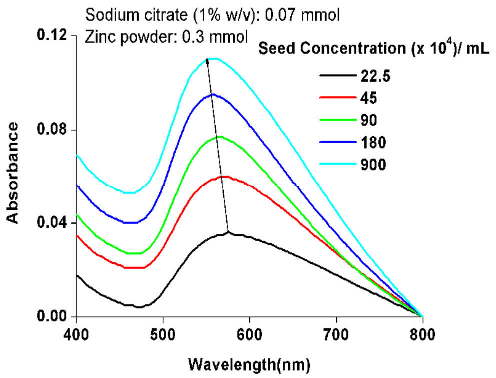

Fig.S1 Absorption spectra of Au-ZnO nanocomposites (**aZn1** to **aZn5**) with seed variation (left) and with zinc variation (right).

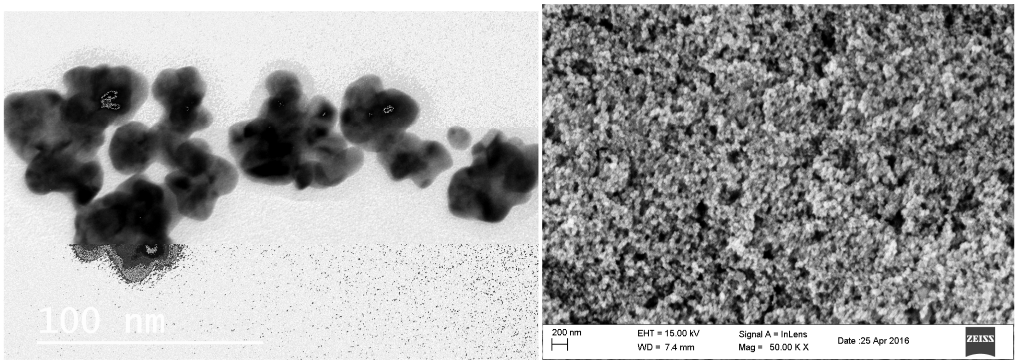

Fig.S2 TEM (left) and FE-SEM (right) image of nanocomposite **aZn5**.

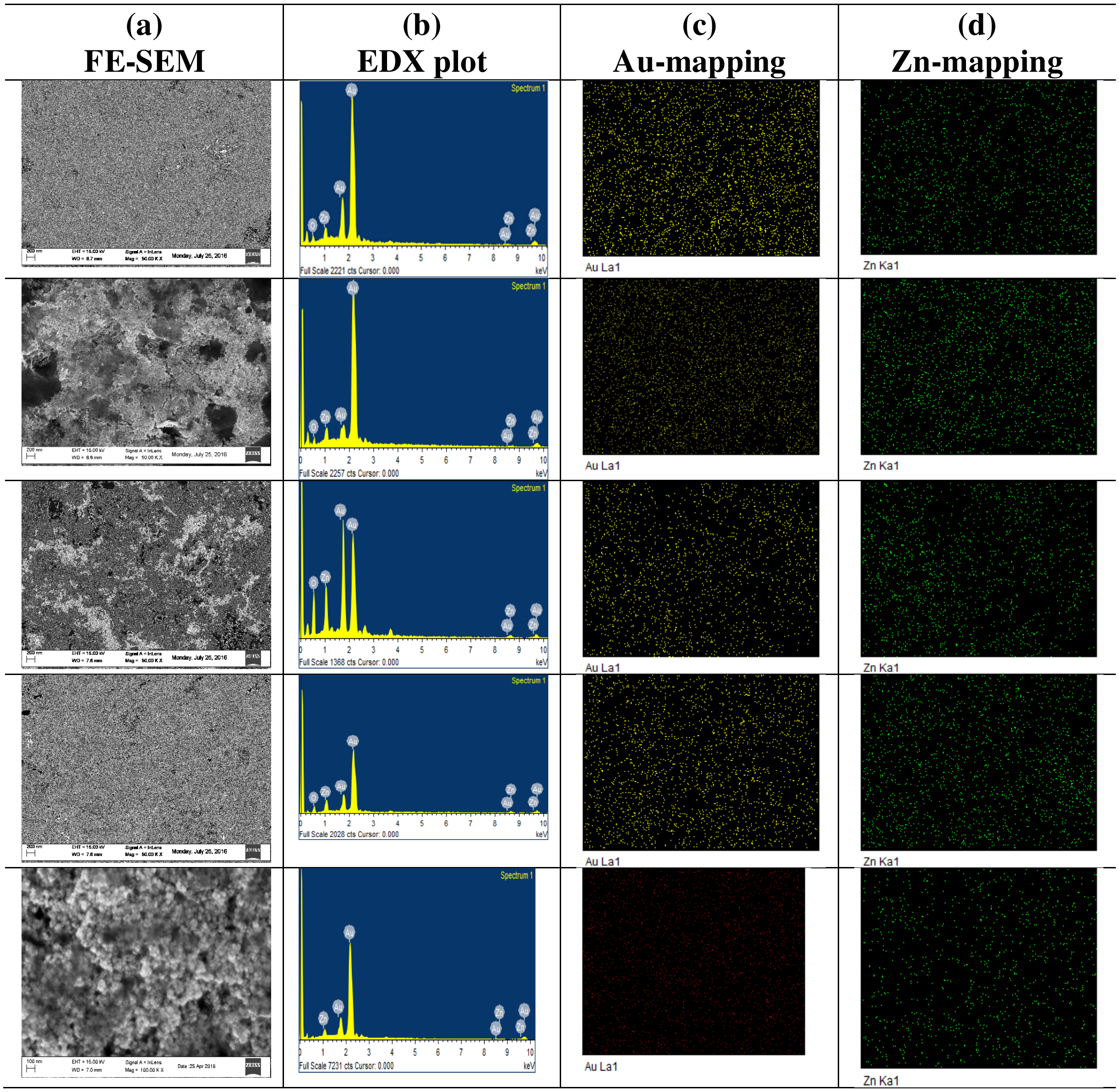

Fig.S3 (a) FE-SEM images, (b) EDX plot (c) EDX mapping of Au and (d) EDX mapping of Zn in nanocomposite **aZn1**-**aZn5** (top to bottom).

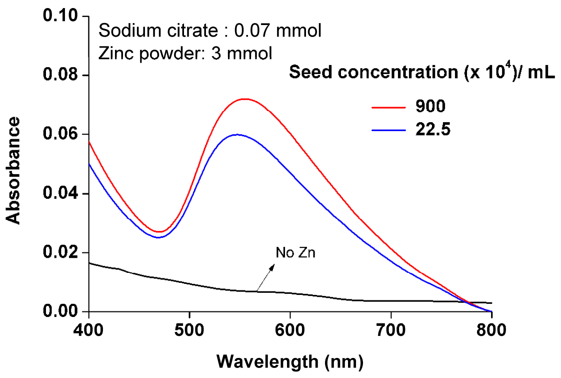

Fig.S4 Absorption spectra of nanocomposites **aZn6** and **aZn7**.

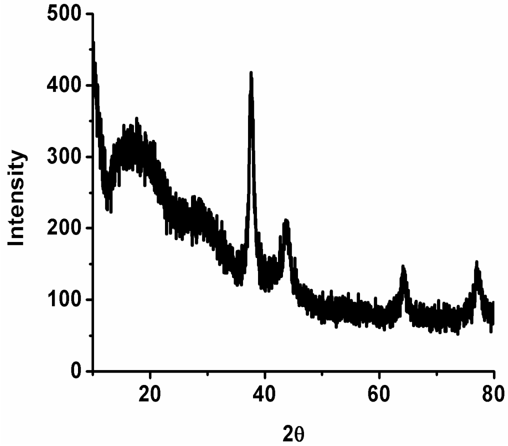

Fig.S5 PXRD of nanocomposites **aZn6**.

(a) FE-SEM	(b) EDX plot	(c) Au-mapping	(d) Zn-mapping
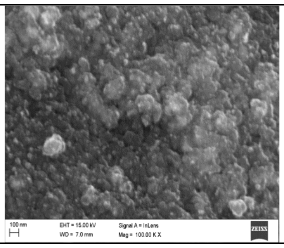	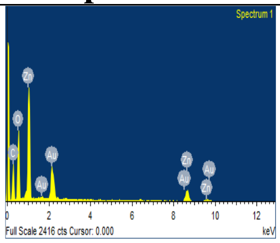	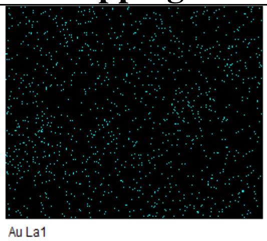	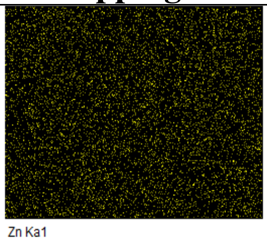

Fig.S6 (a) FE-SEM images, (b) EDX plot (c) EDX mapping of Au and (d) EDX mapping of Zn in nanocomposite **aZn6**.

(a) FE-SEM	(b) EDX plot	(c) Au-mapping	(d) Zn-mapping
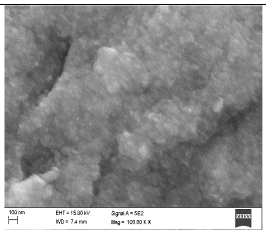	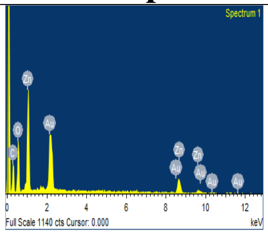	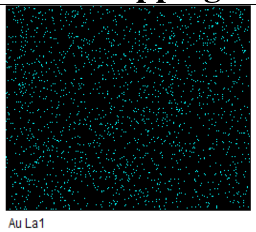	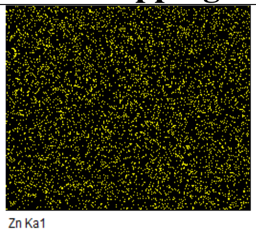

Fig.S7 (a) FE-SEM images, (b) EDX plot (c) EDX mapping of Au and (d) EDX mapping of Zn in nanocomposite **aZn7**.

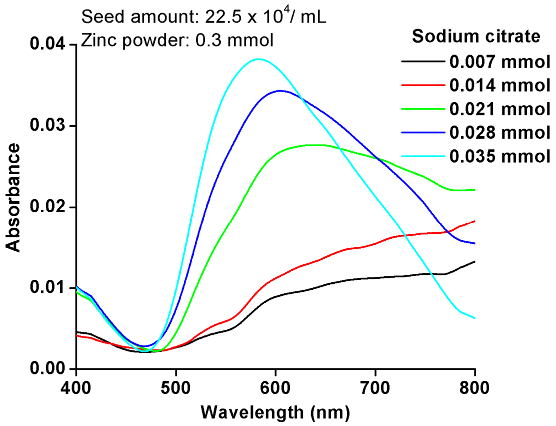

Fig. S8 Absorption spectra of nanocomposites with varying citrate concentration.

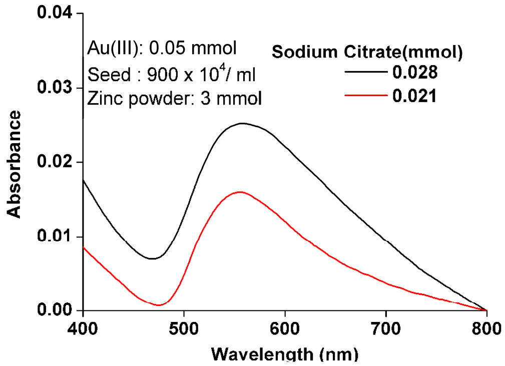

Fig.S9 Absorption spectra of nanocomposites with variable NaCt.

(a) FE-SEM	(b) EDX plot	(c) Au-mapping	(d) Zn-mapping
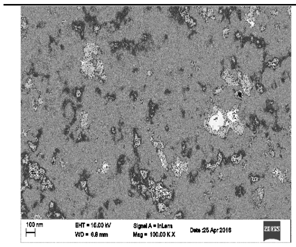	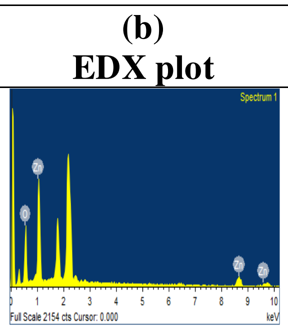	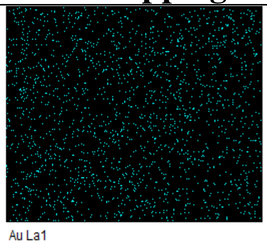	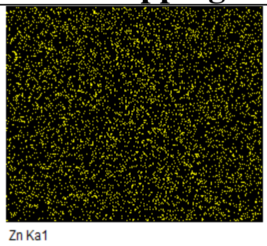

Fig.S10 (a) FE-SEM images, (b) EDX plot (c) EDX mapping of Au and (d) EDX mapping of Zn in nanocomposite **aZn8**.

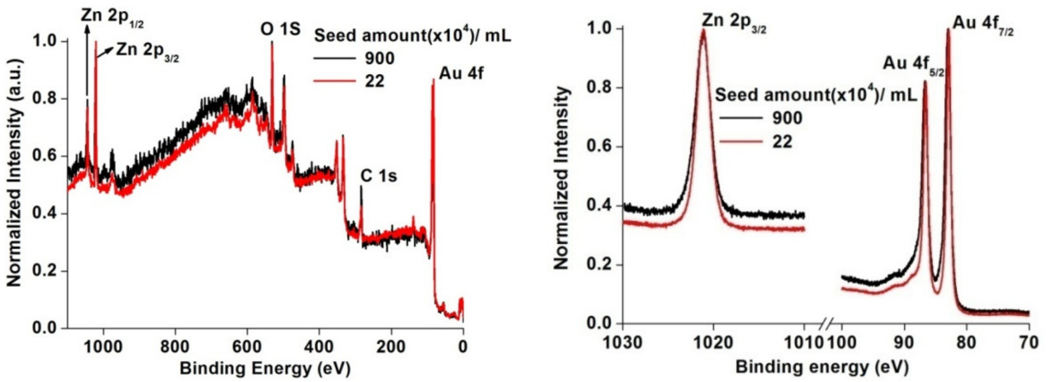

Fig.S11 (Left) survey spectra and (right) the high-resolution Au 4f and Zn 2p XPS spectra of **aZn6** and **aZn7**.

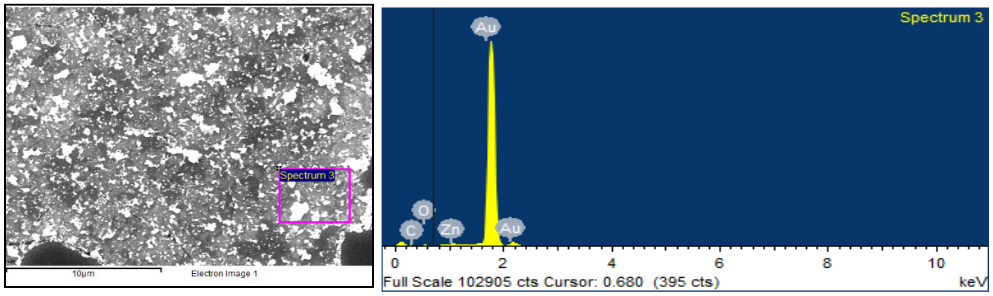

Fig.S12 (Left) FE-SEM images, (right) EDX plot of Au and Zn in nanocomposite **cZn1**.

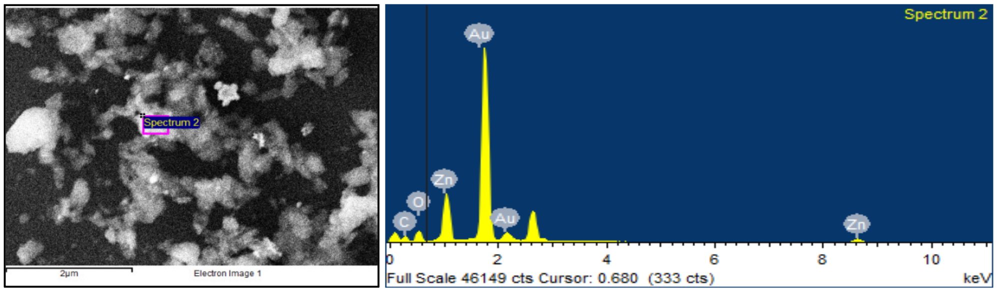

Fig.S13 (Left) FE-SEM images, (right) EDX plot of Au and Zn in nanocomposite **cZn2**.

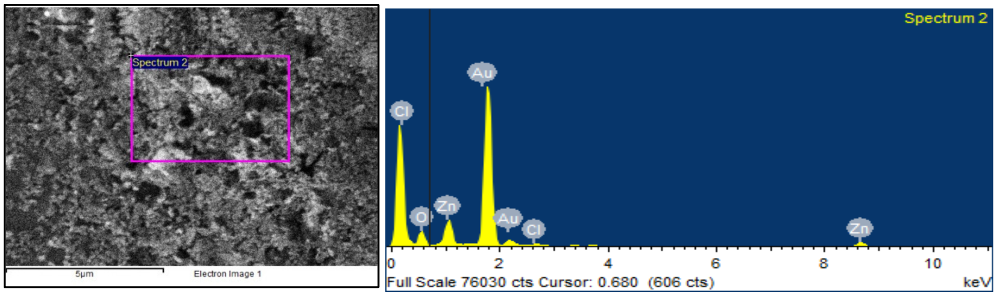

Fig.S14 (Left) FE-SEM images, (right) EDX plot of Au and Zn in nanocomposite **cZn3**.

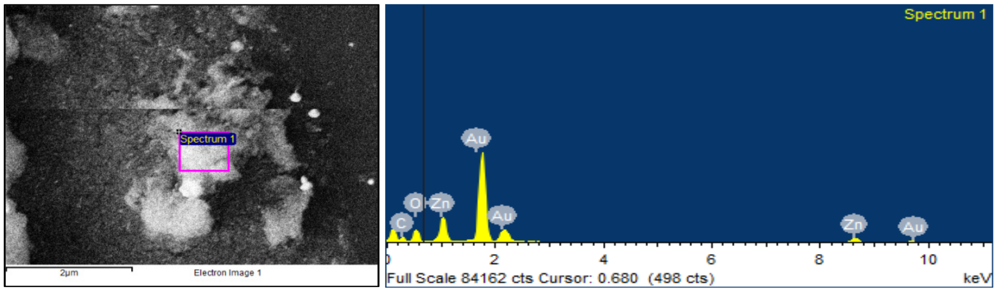

Fig.S15 (Left) FE-SEM images, (right) EDX plot of Au and Zn in nanocomposite **cZn4**.

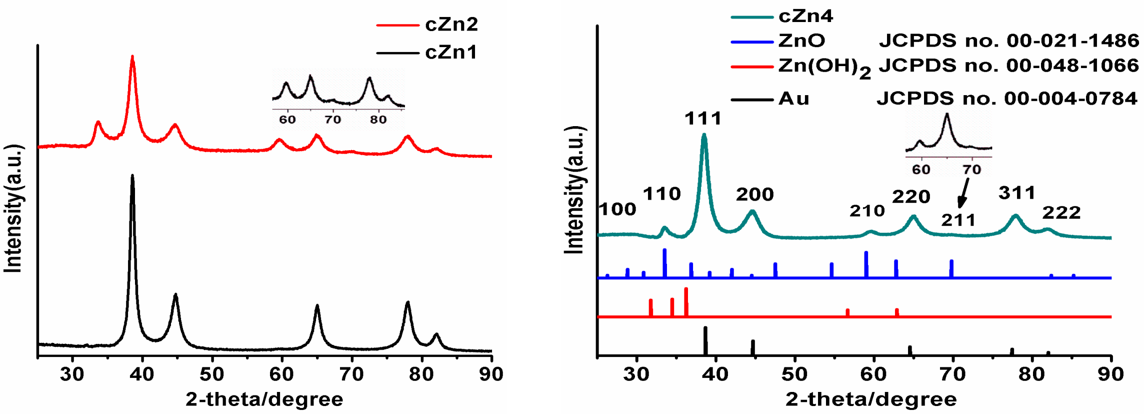

Fig.S16 PXRD of nanocomposites **cZn1**, **cZn2**, **cZn4**

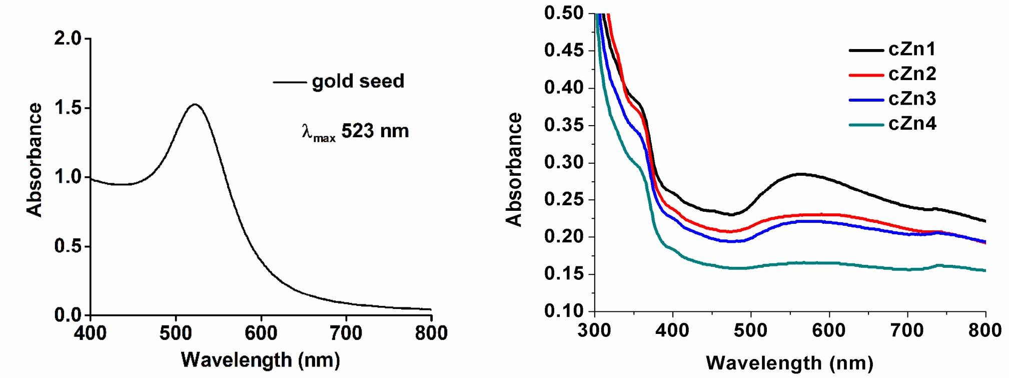

Fig.S17 Absorption spectra of gold nanoparticles (left) and nanocomposites **cZn1**, **cZn2**, **cZn3**, **cZn4** (right)

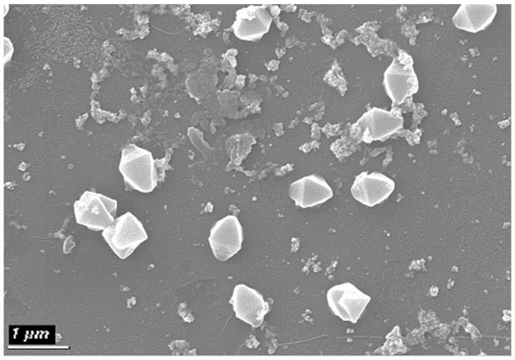

Fig.S18 FESEM image of nanocomposite **cZn1**

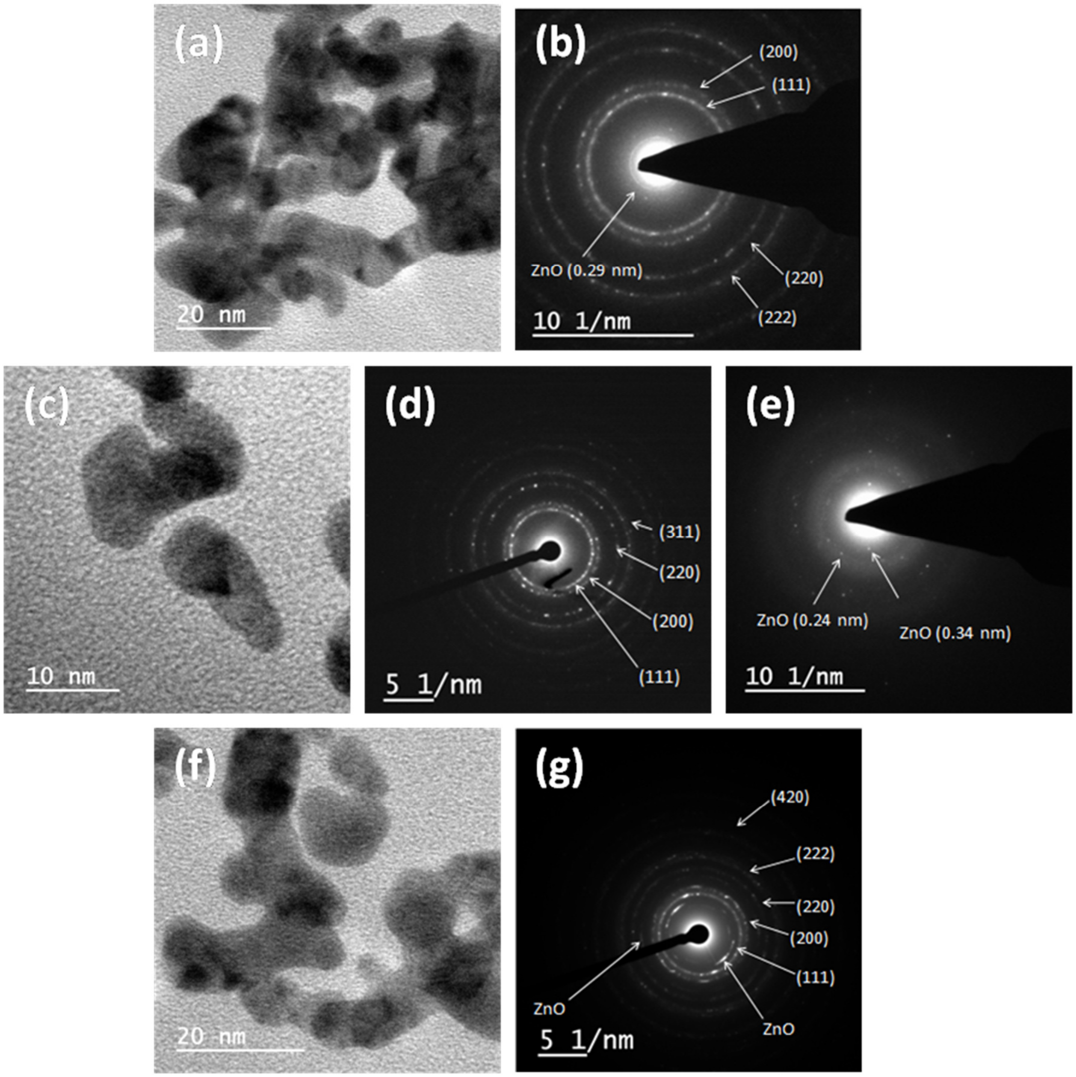

Fig.S19 (a, c, f) TEM images of nanocomposite **cZn1**, **cZn2** and **cZn3** respectively; (b, g) SAED pattern of nanocomposite **cZn1** and **cZn3** respectively showing diffraction pattern corresponding to both Au and ZnO; (d, e) SAED pattern of Au and ZnO respectively for nanocomposite **cZn2**.

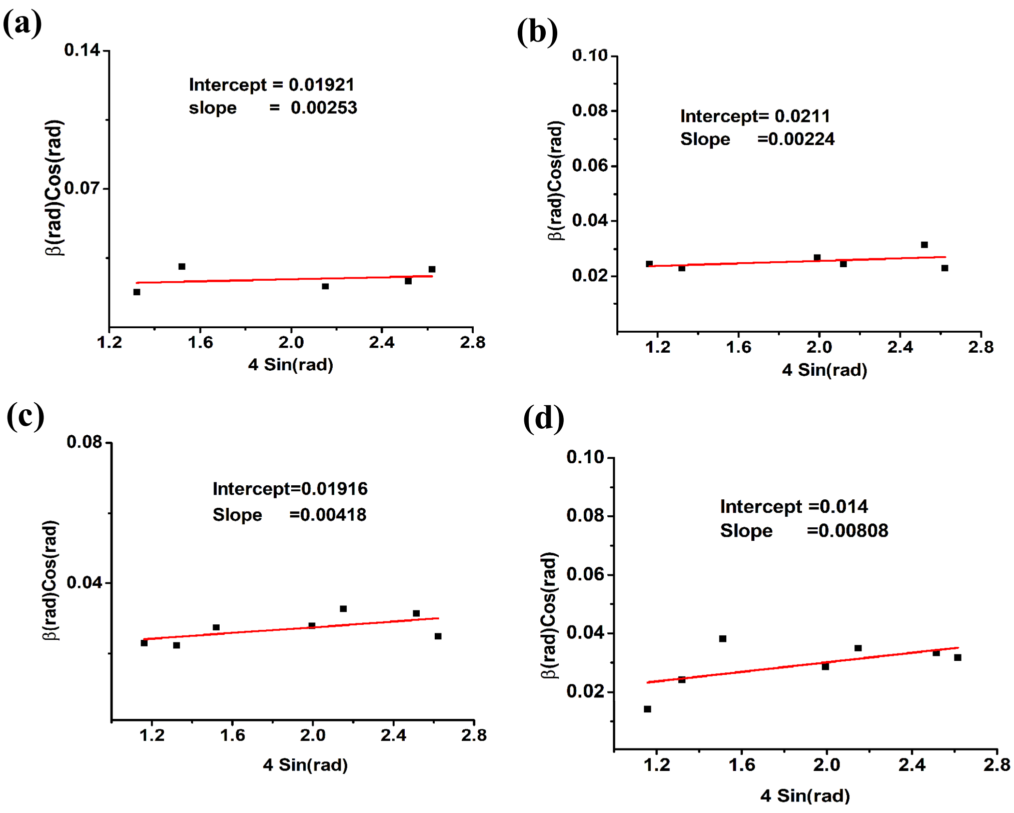

Fig.S20 Williamson Hall Plot of nanocomposite **cZn1, cZn2, cZn3, cZn4**.

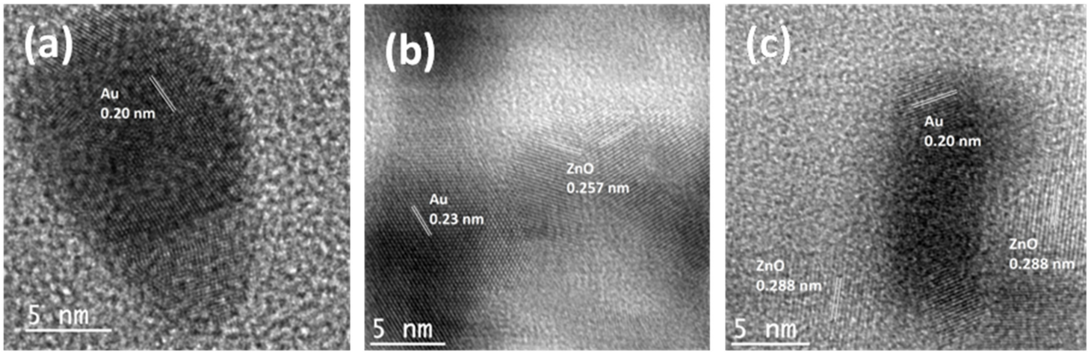

Fig.S21 HRTEM images of nanocomposites (a) **cZn1**, (b) **cZn2** and (c) **cZn3**.

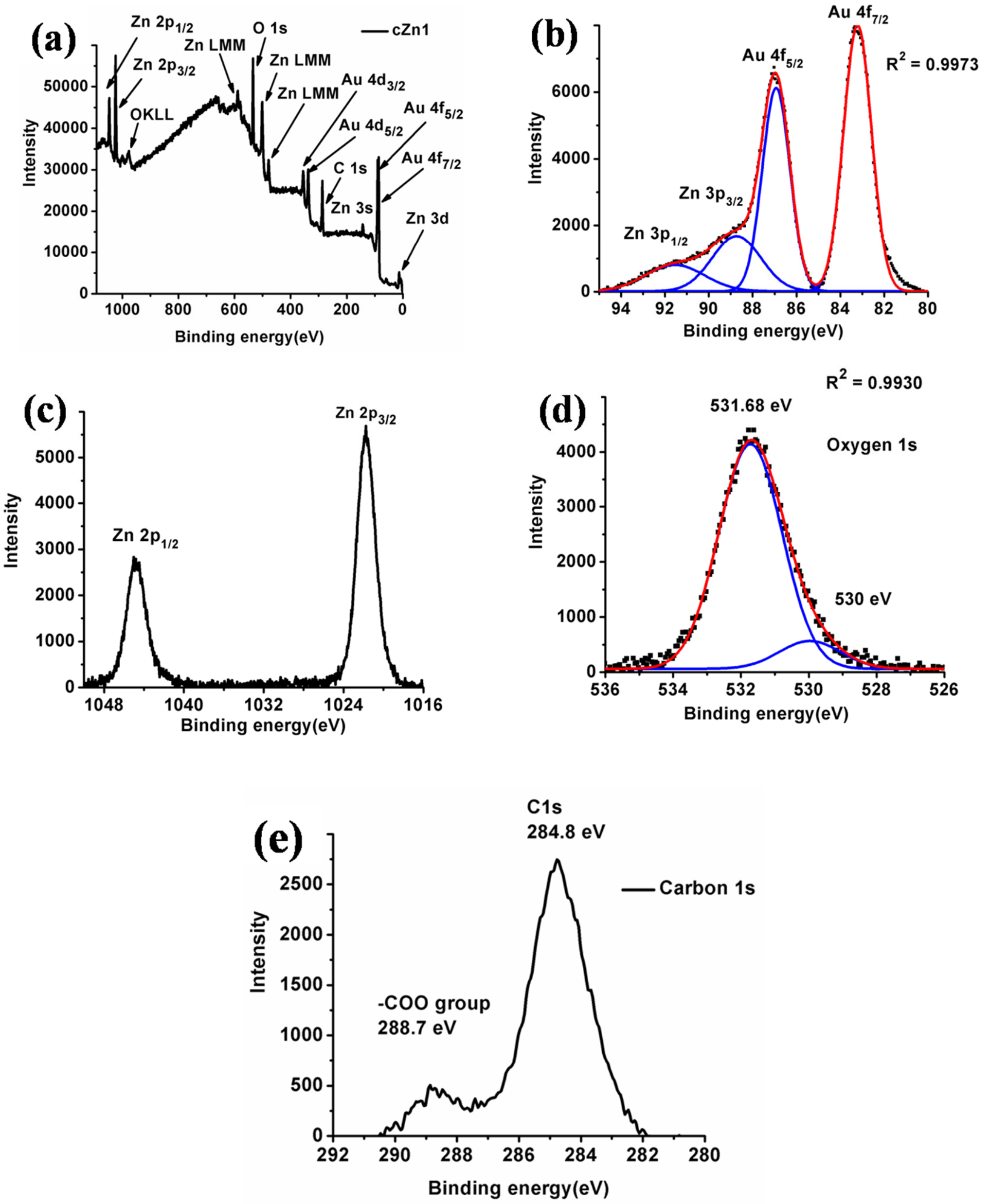

Fig.S22 (a) Survey spectrum of nanocomposite **cZn1**; high-resolution spectra of (b) Au 4f, (c) Zn 2p, (d) O 1s (e) C 1s in nanocomposite **cZn1**.

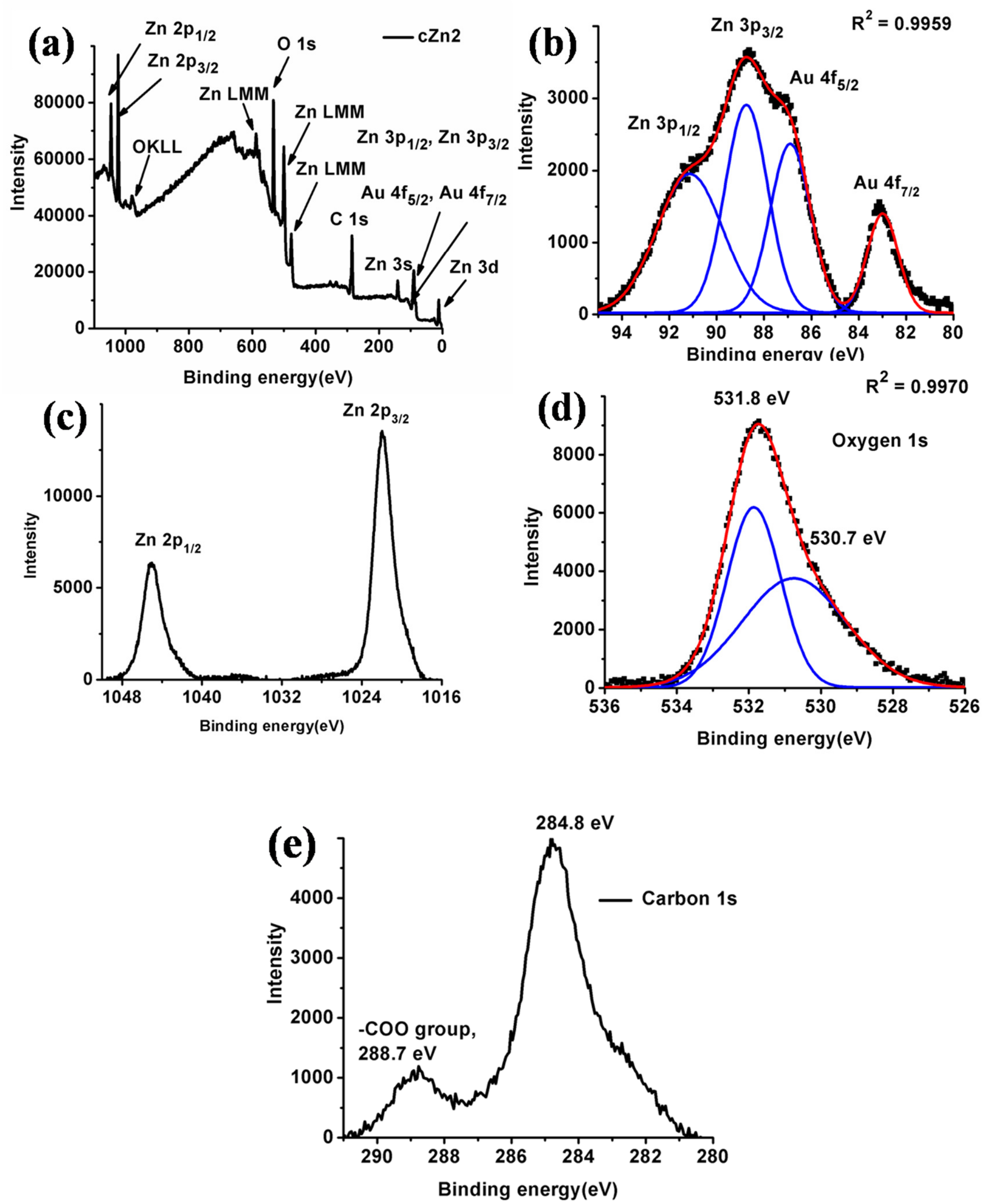

Fig.S23 (a) Survey spectrum of nanocomposite **cZn2**; high-resolution spectra of (b) Au 4f, Zn 3p, (c) Zn 2p, (d) O 1s (e) C 1s in nanocomposite **cZn2.**

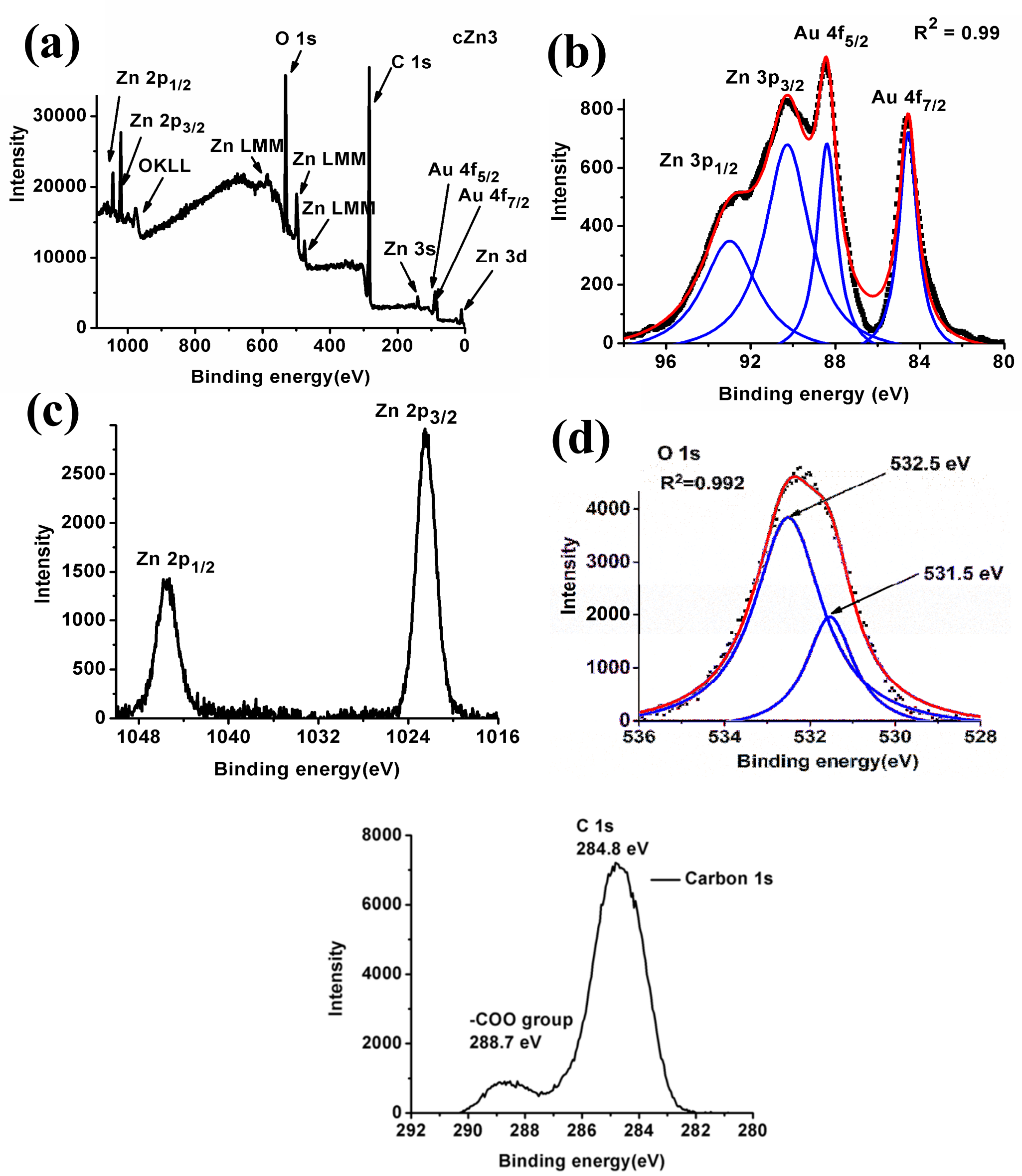

Fig.S24 (a) Survey spectrum of nanocomposite **cZn3**; high-resolution spectra of (b) Au 4f, Zn 3p, (c) Zn 2p, (d) O 1s (e) C 1s in nanocomposite **cZn3**.

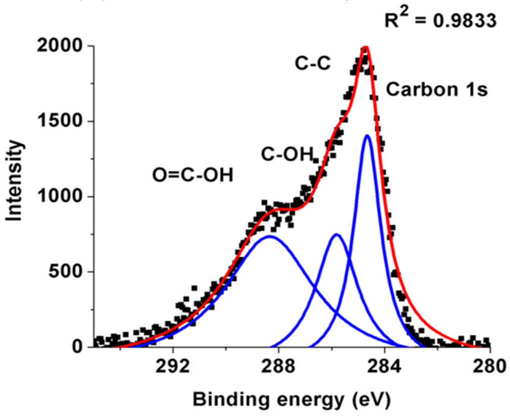

Fig.S25 High resolution XPS spectra of C 1s in **cZn4**

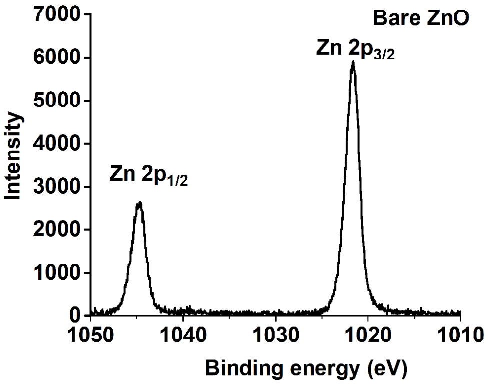

Fig.S26 High resolution XPS spectra of Zn in **cZn4**.

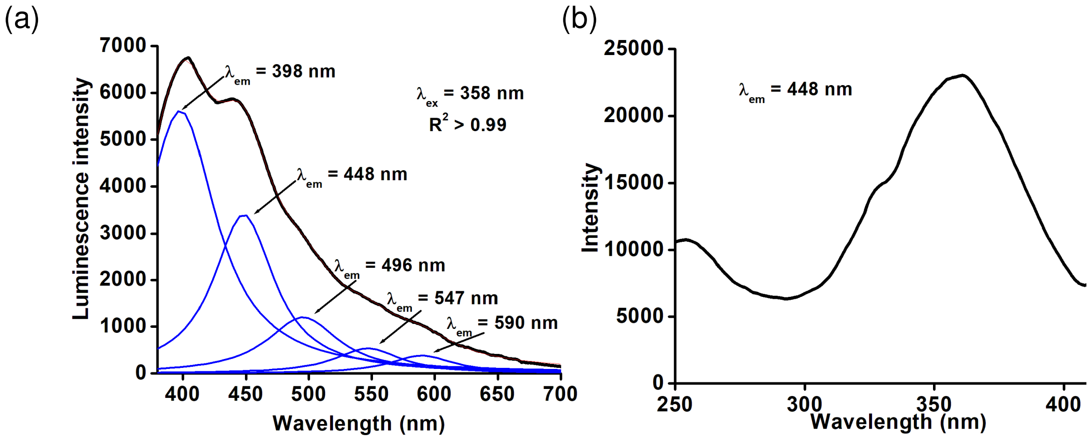

Fig.S27 (a) Emission and (b) excitation spectra of **cZn2**.

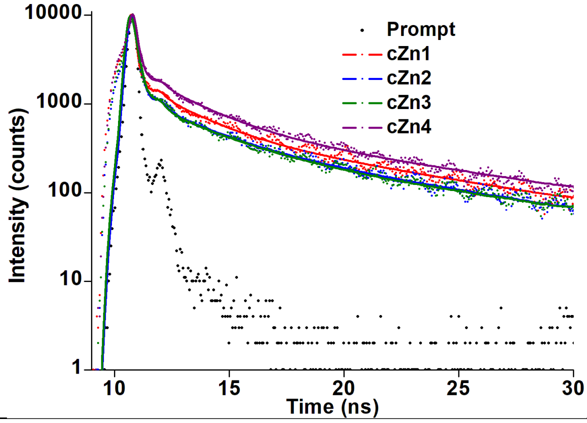

Sample	α_1	τ_1 (ns)	α_2	τ_2 (ns)	α_3	τ_3 (ns)	<τ> (ns)	k_r (s^{-1})	k_{nr} (s^{-1})
**cZn1**	0.26	2.23	0.33	0.12	0.41	11.24	10.17	2.65×10^6	9.57×10^7
**cZn2**	0.23	2.02	0.39	0.10	0.38	12.42	11.40	1.75×10^6	8.60×10^7
**cZn3**	0.27	2.69	0.37	0.17	0.36	13.57	12.03	5.57×10^6	7.76×10^7
**cZn4**	0.19	2.41	0.40	0.22	0.41	9.67	8.76	10.33×10^6	10.33×10^7

Fig.S28 Fluorescence decay plots and data analysis of **cZn1**-**cZn4**.

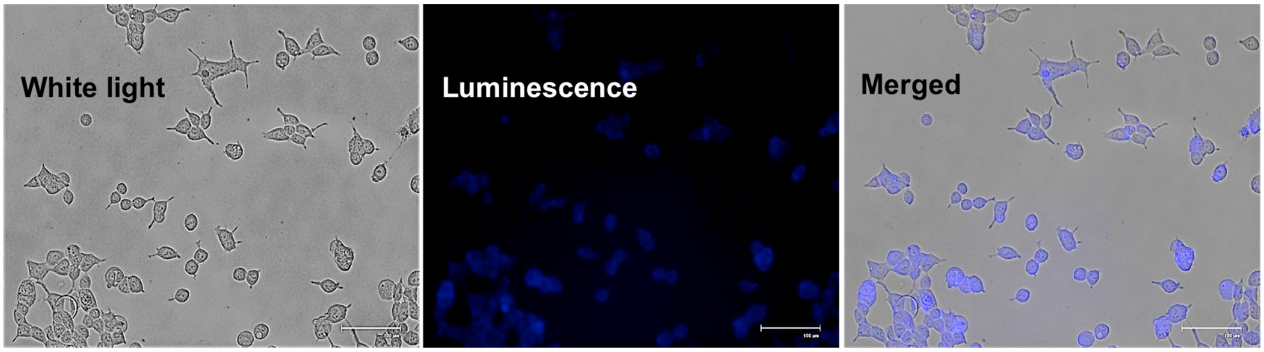

Fig.S29 Imaging of **cZn3** in human embryonic kidney cells (HEK293 cells), where the cells were excited at 390/40 nm and emissions were monitored with 446/33 blue filter for luminescence image, scale bar: 100 μm.

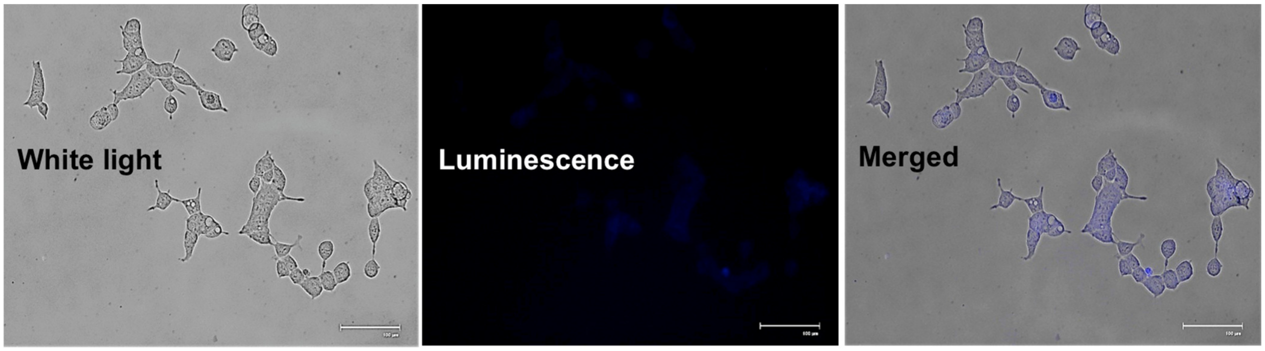

Fig.S30 Imaging of **cZn4** in human embryonic kidney cells (HEK293 cells), where the cells were excited at 390/40 nm and emissions were monitored with 446/33 blue filter for luminescence image, scale bar: 100 μm.

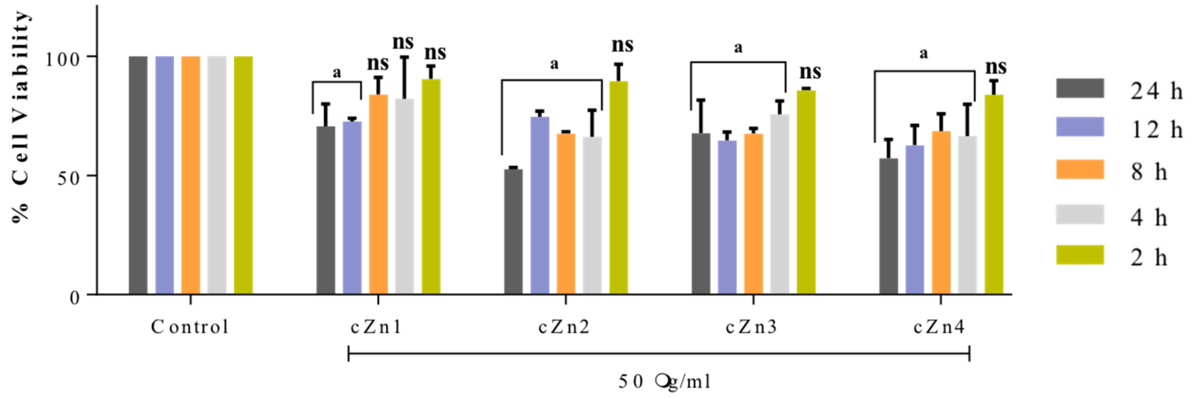

Fig.S31 Cell viability data for human embryonic kidney cells (HEK293 cells) treated with **cZn1**-**cZn4** (50 μg/mL) for different times of incubations. 'a' indicates significantly different with respect to control group ($p<0.05$); ns: non significant

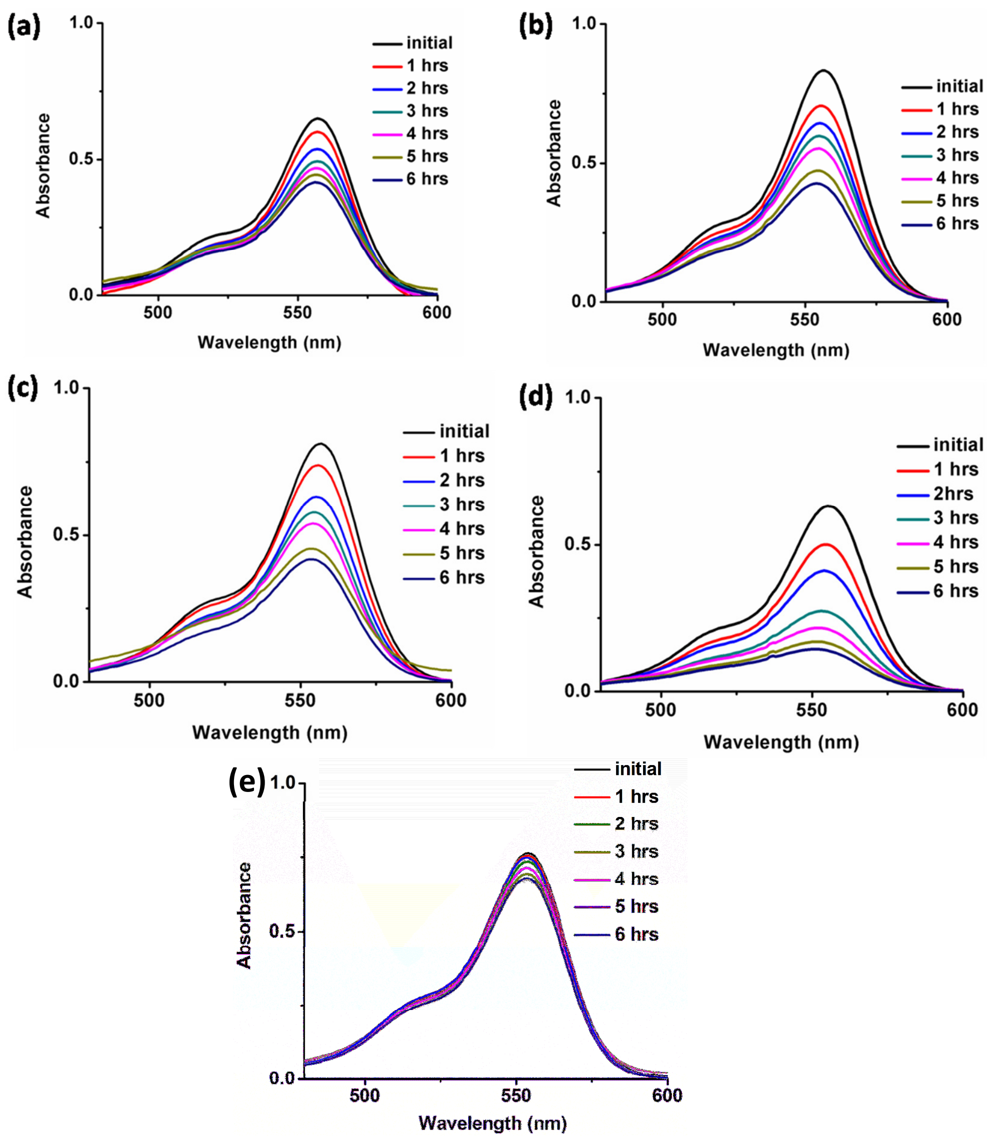

Fig.S32 Absorption spectra taken at regular interval of time showing degradation of rhodamine-B dye upon irradiation with 455 nm LED light in presence of nanocomposites (a) **cZn1**, (b) **cZn2**, (c) **cZn3** and (d) **cZn4** (e) ZnO

Supporting Table

Table S1. XRD peak positions and their corresponding FWHM values (in degrees) are shown in table below for nanocomposites **cZn1**, **cZn2**, **cZn3** and **cZn4**. The peak corresponding to ZnO is highlighted in blue.

cZn1		cZn2		cZn3		cZn4	
Peak position	FWHM	Peak position	FWHM	Peak position	FWHM	Peak position	FWHM
38.57	1.07	33.74	1.46	33.79	1.37	33.61	0.85
44.65	1.90	38.59	1.39	38.60	1.36	38.53	1.48
65.00	1.41	44.58	2.09	44.65	1.70	44.44	2.37
77.95	1.70	59.63	1.77	59.79	1.84	59.78	1.89
81.84	2.23	64.00	1.66	65.07	2.23	64.95	2.37
		77.92	2.33	77.92	2.32	77.87	2.45
		81.87	1.74	81.90	1.88	81.72	2.40

Table S2. XPS peak area ratio of bare ZnO with **Zn1**, **cZn2**, **cZn3** and **cZn4**

	Peak position	Area	Peak area ratio (ZnO:Nanocomposites)
ZnO	1021.6	12379.02	
	1044.7	5143.42	
cZn1	1021	13444.81	0.92:1
	1044.8	7554.60	0.68:1
cZn2	1021.9	36711.21	0.33:1
	1045	18504.81	0.28:1
cZn3	1022.4	6606.14	1.87:1
	1045.5	3532.61	1.46:1
cZn4	1022.9	26149.98	0.47:1
	1045.12	14606.37	0.35:1

Table S3. PDI and Zeta potential of nanocomposite **cZn1**, **cZn2**, **cZn3** and **cZn4**

Nanocomposite	PDI	Zeta potential (mV)
**aZn5**	0.202	-0.53
**aZn6**	0.401	-23.5
**aZn7**	0.250	-27.1
**aZn8**	0.399	-19.7
**cZn1**	0.217	-3.78
**cZn2**	0.235	-0.33
**cZn3**	0.172	-24.4
**cZn4**	0.274	0.36
